# Early Detection of Diabetic Peripheral Neuropathy: A Focus on Small Nerve Fibres

**DOI:** 10.3390/diagnostics11020165

**Published:** 2021-01-24

**Authors:** Jamie Burgess, Bernhard Frank, Andrew Marshall, Rashaad S. Khalil, Georgios Ponirakis, Ioannis N. Petropoulos, Daniel J. Cuthbertson, Rayaz A. Malik, Uazman Alam

**Affiliations:** 1 Diabetes & Endocrinology Research, Institute of Cardiovascular and Metabolic Medicine and The Pain Research Institute, University of Liverpool, Liverpool L69 7ZX, UK; hirkhal2@liverpool.ac.uk (R.S.K.); Dan.Cuthbertson@liverpool.ac.uk (D.J.C.); 2 The Walton Centre, Department of Pain Medicine, Liverpool L9 7LJ, UK; Bernhard.Frank@thewaltoncentre.nhs.uk; 3 Department of Musculoskeletal & Ageing Science, Faculty of Health & Life Sciences, Institute of Life Course & Medical Sciences, University of Liverpool, Liverpool L7 8TX, UK; Andrew.Marshall@liverpool.ac.uk; 4 Faculty of Health and Life Sciences, The Pain Research Institute, University of Liverpool, Liverpool L9 7AL, UK; 5 The Walton Centre, Department of Clinical Neurophysiology, Liverpool L9 7LJ, UK; 6 Weill Cornell Medicine-Qatar, Qatar Foundation, Education City, Doha P.O. Box 24144, Qatar; g.ponirakis@gmail.com (G.P.); inp2002@qatar-med.cornell.edu (I.N.P.); ram2045@qatar-med.cornell.edu (R.A.M.); 7 Institute of Cardiovascular Sciences, University of Manchester, Manchester M13 9PL, UK; 8 Division of Endocrinology, Diabetes and Gastroenterology, University of Manchester, Manchester M13 9PT, UK

**Keywords:** diabetes, neuropathy, peripheral neuropathy, distal sensory polyneuropathy, diabetic neuropathy, diabetic peripheral neuropathy, early detection, screening, diagnostics, point-of-care

## Abstract

Diabetic peripheral neuropathy (DPN) is the most common complication of both type 1 and 2 diabetes. As a result, neuropathic pain, diabetic foot ulcers and lower-limb amputations impact drastically on quality of life, contributing to the individual, societal, financial and healthcare burden of diabetes. DPN is diagnosed at a late, often pre-ulcerative stage due to a lack of early systematic screening and the endorsement of monofilament testing which identifies advanced neuropathy only. Compared to the success of the diabetic eye and kidney screening programmes there is clearly an unmet need for an objective reliable biomarker for the detection of early DPN. This article critically appraises research and clinical methods for the diagnosis or screening of early DPN. In brief, functional measures are subjective and are difficult to implement due to technical complexity. Moreover, skin biopsy is invasive, expensive and lacks diagnostic laboratory capacity. Indeed, point-of-care nerve conduction tests are convenient and easy to implement however questions are raised regarding their suitability for use in screening due to the lack of small nerve fibre evaluation. Corneal confocal microscopy (CCM) is a rapid, non-invasive, and reproducible technique to quantify small nerve fibre damage and repair which can be conducted alongside retinopathy screening. CCM identifies early sub-clinical DPN, predicts the development and allows staging of DPN severity. Automated quantification of CCM with AI has enabled enhanced unbiased quantification of small nerve fibres and potentially early diagnosis of DPN. Improved screening tools will prevent and reduce the burden of foot ulceration and amputations with the primary aim of reducing the prevalence of this common microvascular complication.

## 1. Introduction

The International Diabetes Federation (IDF) estimated the global prevalence of diabetes is 425 million people in 2017 and is predicted to rise to 628 million by 2045 [1]. This has been accompanied by an increase in the burden of diabetic complications [2,3]. Diabetic neuropathy affects 10–50% of people with type 1 (T1D) and type 2 diabetes mellitus (T2D) [4,5,6,7]. In the US, the annual cost for managing DPN and foot ulceration with lower limb amputation is estimated to be between $4.6–13.7 billion [8]. Diabetic peripheral neuropathy (DPN) has a predilection for small unmyelinated or thinly myelinated C and Aδ nerve fibres [9], which mediate temperature and pain perception, tissue blood flow and sweating, all of which are key factors for foot ulceration [10]. Small fibre deficits are considered to precede large fibre involvement in DPN [5,11]. Furthermore, small fibre degeneration occurs in prediabetes suggesting early subclinical pathology before the onset of overt T2D [12,13]. Indeed, small fibres are the earliest to degenerate and have the greatest potential for repair as shown in studies with normalisation of hyperglycaemia through pancreatic transplantation in T1D and weight loss following lifestyle intervention in prediabetes [14,15,16].

### 1.1. Economic and Functional Consequences of Small Fibre Degeneration

Degeneration of small sensory nerve fibres occurs in painful DPN (pDPN) which is present in up to one-third of patients with diabetes [17,18,19]. Neuropathic pain has a profound impact on quality of life, physical and emotional health and affects both functionality and sleep [20,21,22]. Chronic intractable pain is associated with anxiety and depression and is often refractory to current therapies [21,23]. Consequently, people with pDPN are more likely to be unemployed and loss of working time in a U.S. population cost ~$3.65 billion each year [24,25]. Furthermore, people suffering severe chronic pain have an increased ten-year mortality [26].

DPN is significantly underdiagnosed leading to missed opportunities for preventing progression to severe DPN and foot ulceration, which has a dreadful 5-year mortality [27,28,29]. Indeed, DPN is a major cause of foot ulceration and is implicated in 50–75% of all non-traumatic amputations [30,31]. Mortality, one and five-years after lower limb amputation in people with diabetes ranges from 10–50%, to 30–80% respectively [32,33,34] with the latter mortality rate comparable to lung cancer [35]. DPN and amputation represent a devastating impact on the individual leading to a loss of function, quality of life and financial stability [36,37]. In the UK, the National Health Service (NHS) spent £639 million on diabetic foot ulcers and £662 million on lower limb amputations, accounting for £1 in every £150 spent out of the NHS healthcare budget [38].

### 1.2. Pathogenesis

Whilst there has been progress in identifying the pathophysiology of DPN, a complete understanding of this process remains elusive [39]. DPN is associated with hyperglycaemia, hyperlipidaemia, insulin resistance and protein catabolism [6,40]. Hyperglycaemia-induced oxidative stress and reactive oxygen species result in peripheral nerve injury [41,42]. Experimental data have demonstrated nitro-oxidative stress in dorsal root ganglia, axons and Schwann cells with nerve conduction impairment, neurovascular dysfunction, apoptosis and sensory deficits [43,44,45,46]. There is also activation of poly (ADP-ribose) polymerase, polyol, hexosamine and protein kinase C (PKC) pathways and accumulation of advanced glycation end products culminating in axonal dysfunction and damage [44,47,48,49,50,51]. Increased flux through the polyol pathway leads to accumulation of sorbitol and fructose, myo-inositol depletion and a reduction in Na^+^K^+^-ATPase activity. Endoneurial microvascular deficits result in hypoxia and ischaemia, generation of reactive oxygen species (oxidative stress), activation of the redox-sensitive transcription factor NFκB, and increased activity of PKC [52,53].

### 1.3. Evidence in Favour of Early Intervention for DPN

The incidence of DPN is associated with hyperglycaemia and also cardiovascular risk factors such as raised cholesterol, triglycerides, hypertension, obesity and smoking [54]. Indeed, all of these risk factors can be modulated by early intervention. In a large longitudinal study of 1441 people with T1D in the Diabetes Control and Complications trial (DCCT), intensive insulin treatment reduced the risk of developing DPN by 60% [55,56]. In fact, a continuous beneficial effect after intensive insulin treatment was observed in participants of the DCCT trial after 10 years of follow-up in the Epidemiology of Diabetes Interventions and Complications (EDIC) trial [57]. Furthermore, a Cochrane Systematic Review found enhanced glucose control significantly reduced the risk of developing DPN in participants with T1D compared to standard of care [58]. However, whilst tight glucose control reduces the incidence of DPN in T2D, this reduced risk was not statistically significant [58] and in T2D, DPN has greater multifactorial causality due to the heterogeneous nature of the disease. For instance, obesity and hypertriglyceridemia are significant risk factors of DPN for people with T2D, independent of glucose control [59]. It follows that treatment of hypertension in people with T2D is associated with a significant reduction in the incidence of DPN and improvements in people with mild DPN [60,61,62]. Furthermore, a small randomised, double-blind, placebo-controlled Phase IIa study of participants with T2D and early DPN, found that reduction of low-density lipoprotein (LDL) cholesterol and triglycerides using rosuvastatin improved the neuropathy score and nerve conduction parameters [63]. Individualised diet and aerobic and resistance exercise regimens are important in the reversal of early DPN changes and the prevention of progression to DPN [15,64,65]. Thus, a multifactorial approach is required for the prevention and early treatment of DPN of people with T2D. A key underpinning of multifactorial treatment is an accurate, reiterative diagnostic modality for the screening of people with diabetes to reliably detect early DPN.

### 1.4. Current Clinical Assessment of Neuropathy

The signs and symptoms of DPN are insidious and current screening programmes rely on subjective tests of large nerve fibre dysfunction [66,67]. NICE recommends vibration perception testing using a 128 Hz tuning fork together with a 10 g (Semmes-Weinstein) monofilament for the screening of DPN [67]. However, these tests identify DPN at a late, irreversible, pre-ulcerative stage [68,69,70]. Thus, an abnormal monofilament test is associated with a 3-year relative risk of 15% (95% CI 9.0 to 26.0) for foot ulceration or lower limb amputation [71]. Despite, early and progressive injury to small fibres in diabetes, small nerve fibre assessment is not included in annual diabetic foot screening programmes [11,72].

In direct contrast, diabetic retinopathy and diabetic kidney disease have effective screening programmes which detect early sub-clinical pathology, enabling early interventions [73] which has led to a reduction in blindness [73] and end stage renal failure [74]. In fact, largely due to the success of diabetic retinopathy screening, it is no longer the leading cause of sight loss in western society [75]. Early, multifactorial risk factor modification may reduce the risk of foot ulceration and amputation [76,77]. The Toronto Diabetic Neuropathy Expert Group and American Diabetes Association (ADA) [6,78] have recommended the early detection and monitoring of DPN, but have recommended monofilament or crude neurological testing. Clearly, there is a need for robust screening methods capable of diagnosing subclinical DPN. This article aims to critically appraise commonly used research and clinical diagnostic tools to evaluate their potential role in screening for early DPN.

### 1.5. Methods

Electronic database searches were undertaken in Google Scholar, EMBASE, PubMed, OVID and Cochrane CENTRAL to identify the included articles. Reference lists of relevant articles were searched and in addition, studies were identified by authors with expertise in DPN. Studies published from initial curation of the electronic database to November 2020 were identified and those felt not relevant by authors were excluded with the guidance of the senior author (U.A.).

## 2. Screening and Diagnostic Tools

### 2.1. Composite Scoring Systems

Confirmed DPN represents a subtle and gradual disease process, in which the symptoms are often unreliable as an indicator of early nerve damage [79,80]. However, neuropathic pain may be the initial presenting symptom of diabetic neuropathy in patients with diabetes, pre-diabetes or metabolic syndrome [8]. Thus, validated screening instruments which utilise sensory and affective verbal pain descriptors (burning sensation, tingling/prickling, numbness, electric shocks, pain evoked by light touch) such as the Leeds Assessment of Neuropathic Symptoms and Signs (LANSS), douleur neuropathique en 4 (DN4) and painDETECT are widely used for the identification of neuropathic pain in diabetes [9,10,11,12,13,14]. Notably, Bennet et al. [15] demonstrated the utility of screening for neuropathic pain in a population of people with diabetes using a postal self-completed portion of the LANSS questionnaire, highlighting the excess prevalence and burden of pDPN. However, measures such as the neuropathy symptom score (NSS) cannot reliably identify early DPN [81,82]. Numerous clinical scoring systems have been compiled to evaluate light touch, pin-prick, vibration, proprioception, muscle strength and ankle reflexes [83]. The Michigan neuropathy screening instrument (MNSI) evaluates both positive and negative sensory symptoms and an examination of the foot to identify dry skin and ulcers [84]. In a large cohort of 1100 people with T1D Herman et al. [85] showed that the MNSI questionnaire had a low sensitivity, high specificity and only a moderate negative predictive value (NPV) compared to nerve conduction studies (NCS). Furthermore, the MNSI questionnaire was unable to identify the development of DPN over 13 years in a cohort of 1256 participants with T2D in the ACCORD-Denmark study [86]. VPT and NCS identified a higher prevalence of DPN compared to the MNSI and physical examination in a cohort of participants with T2D [87].

The neuropathy disability score (NDS) compiled by Dyck et al. [81] is a composite scoring system that assesses signs of neuropathy. A simplified NDS has been utilised widely to identify the signs and severity of DPN using a 0–10 scoring system [88], with a score of ≥6 to define established neuropathy [89] and can also be used to stratify patients into mild (3–4), moderate (5–6) or severe neuropathy (7–10) or those at high risk of foot ulceration as it is weighted for large fibre testing [90].

The neuropathy impairment score (NIS) has been adapted to assess DPN by focusing the examination to the lower limbs (NIS-(LL)) incorporating an assessment of muscle weakness, reflexes and sensory loss [91]. The NIS-LL+7 includes the NIS and seven additional tests including five different NCS of the lower limbs, vibration detection and heart rate response to deep breathing. NIS-LL+7 has demonstrated the capacity to identify early changes in DPN and worsening in a longitudinal study [82]. However, the inclusion of multiple tests into a single composite score is labour intensive and time consuming and is not suitable for screening [92]. These composite scoring systems and the current annual diabetic foot check are not effective in the detection of early DPN.

### 2.2. Thermal and Vibration Perception Thresholds

The detection of sub-clinical neuropathy in both symptomatic and asymptomatic patients with diabetes mellitus in the early stages of the disease is key to providing a window of opportunity for optimising multifactorial treatment and limit the progression of DPN [93]. The routine use of thermal thresholds in clinical practice has been difficult to implement due to the cost and subjective nature of the testing and a lack of consensus on standard practice [94,95]. Abnormal thermal sensation has been identified in over 93% of subjects with impaired glucose tolerance (IGT) or T2D [96] and in ~50% of asymptomatic participants with diabetes and normal NCS [97].

A large cross-sectional study found participants with T2D had a higher heat detection threshold (HDT) and lower cold detection threshold (CDT) of both the feet and hands compared to healthy volunteer control participants [98]. Moreover, in participants with T2D the prevalence of abnormal thermal thresholds in the big toe (60.2%) and on the dorsum of the foot (45.2%) was higher than abnormalities in sural nerve action potential (SNAP) (12.9%) [98]. Thermal threshold abnormalities are also present in participants with early-onset T2D with further abnormalities in painful and painless DPN compared to controls [99,100,101]. Interestingly, thermal threshold testing outperformed intra-epidermal nerve fibre density (IENFD) in the detection of an abnormality (abnormal thermal thresholds: 50% vs. reduced IENFD: 40%) in a cohort of 210 participants with signs and symptoms of neuropathy [102]. Detecting sensory abnormalities using VPT, pinprick, thermal thresholds and light touch testing in participants with confirmed neuropathy has demonstrated high specificity (77–96%) and positive predictive value (PPV) (95–98%) for DPN [103]. Thus, thermal thresholds and pinprick testing may be more useful in identifying early DPN as they test C and Aδ fibres whilst VPT and light touch are measures of large Aβ fibre function [104,105,106,107]. The results of VPT, pinprick and light touch testing modalities using a standardised protocol have demonstrated good reliability in healthy controls and people with DM [108,109,110]. However, a wide range of variability has been reported across studies ranging from poor to excellent for cold detection threshold (CDT), heat detection threshold (HDT), cold pain threshold (CPT) and heat pain threshold (HPT) [111,112].

### 2.3. German Network on Neuropathic Pain

The protocol developed by the German Research Network for Neuropathic Pain (DFNS) is comprised of the following components; mechanical detection threshold (MDT), mechanical pain threshold (MPT), C-tactile afferents [113], wind-up ratio (WUR) [114], pressure pain threshold (PPT), vibration detection threshold (VDT), thermal thresholds (TT) and thermal sensory limen (TSL) [115]. The loss of small nerve fibre sensitivity or gain of function can be detected using QST for different nerve fibre populations [116,117]. The DNFS have published age and sex matched normative data for discrete areas of the trunk, face, hand and foot for participants aged 6–75 years [118,119,120]. The DNFS QST static protocol differs from bed-side sensory testing through standardisation of the stimuli and standardised instructions to the patient and examiners to execute the static protocol [121,122]. Dynamic QST assesses the change in sensitivity of a test before and after a painful stimulus to identify mechanisms of pain processing as opposed to the static QST limen which measure the basal states of the nociceptive system [115]. Whilst no reference data are currently available for dynamic QST, practical recommendations have been published [123].

Notably, Kopf et al. [124] identified sensory abnormalities in 71% of participants with pre-diabetes, and 95% of participants with T2D, outperforming NCS. Further, participants with T2D had greater deficits in large fibre function and CDT with a gain of function in both MPT and PPT compared to pre-diabetes [124]. Correspondingly, a large study of a cohort of 350 participants with polyneuropathy found that participants with small fibre neuropathy had a loss of Aδ and C-fibre function (CDT, WDT and TSL; all *p* < 0.001) whilst participants with polyneuropathy had loss of function in both small and large fibres (CDT, WDT and TSL; all *p* < 0.001, MDT and VPT; both *p* < 0.001) compared to 273 controls [125].

Maier et al. [110] showed the suitability of QST for identifying sensory abnormalities in 1200 participants with a wide range of polyneuropathies as there was at least one sensory abnormality in 92% of all patients with neuropathy. Interestingly, IENFD can be significantly decreased despite, normal sensory detection thresholds [126]. In a recent criticism, Schmelz [127] argues that QST is unable to differentiate painful from non-painful neuropathies [128]. Importantly, the German DNFS QST protocol does not directly differentiate between central nervous system or peripheral nerve involvement [129], but may be used as a supplementary diagnostic modality characterising sensory phenotypes [122,130,131]. Thus, the combination of QST together with another functional or structural measure of neuropathy such as NCS, IENFD or corneal confocal microscopy (CCM) is suggested [12,132]. QST may be useful to identify patients who may have small fibre deficits, especially asymptomatic patients with normal NCS [133,134]. A large multi-centre study found low heterogeneity across the full static DNFS QST protocol [135]. Environmental and methodological factors and the capacity of the participant to interact appropriately with the QST testing protocol are pivotal to the quality assurance of QST [112,122], but is reliable and reproducible in generating sensory phenotypes.

### 2.4. Evoked Potentials

The European Federation of the Neurological Societies (EFNS) have identified evoked potentials as a non-invasive, reliable tool for investigating Aδ fibre function in patients with neuropathic pain [131]. Laser evoked potentials (LEPs) have been identified as “grade A” evidence by the EFNS [131]. The response of primary nociceptive afferents to LEPs and contact heat evoked potentials (CHEPs) assesses the activation of the primary and secondary somatosensory cortex, insula and mid-cingulate cortex [136,137]. LEPs are elicited by skin stimulation with afferent radiant heat emitted by a CO_2_ or solid-state laser [138,139]. A𝛿-fibre function is recorded through scalp electrodes measured at a late range latency of the brain action potential (200–400 ms) [116]. Age-corrected normative reference ranges from LEPs have been published from healthy controls and validated against IENFD [140]. Although normative data has been published, no consensus has been reached on CHEP methodology, cautioning against the use of these normative values if the methodology is different for eliciting CHEPs [141,142,143,144]. Further, CHEP amplitudes decrease with age in healthy control subjects and vary between male and female participants [142,143,145].

Differences between participants with polyneuropathy and controls have been identified using CHEPS [146,147,148]. Reduced CHEPS amplitudes (prolonged N2 latencies) were identified in 61.2% of patients with symptoms of a length dependent somatosensory neuropathy detecting a greater prevalence of abnormalities than thermal thresholds and NCS [141,149]. In a cross-sectional cohort study, both LEPs and CHEPs latency were reduced in participants with mixed-fibre polyneuropathy, but in participants with small fibre pathology only CHEPS were reduced compared to controls [150].

A small cohort study by Ragé et al. [151] identified a significant reduction in LEP amplitude in participants with T2D compared to T1D (*p* = 0.022) and healthy controls (*p* = 0.027). Similarly, Di Stefano et al. [140] found that LEPs were significantly reduced in participants with DM and hyperalgesia compared to participants with hypoalgesia (*p* < 0.05) [140]. Further, participants with reduced LEPs had a significantly reduced IENFD. Notably, reduced LEPs in the lower limb have been identified in people with diabetes and small fibre neuropathy [133,151]. In a large cross-sectional study by Wu et al. [141] 188 participants with neuropathy had significantly reduced CHEP amplitudes and longer N2 latencies compared to controls (21.7 ± 14.8 vs. 33.8 ± 12.1 µV, *p* < 0.001; 527.4 ± 7.4 vs. 495.1 ± 41.4 milliseconds, *p* < 0.001, respectively). CHEPS has a greater diagnostic efficiency compared to HDT thresholds on the dorsum of the foot (*p* = 0.036) [141]. CHEPS shows good sensitivity (78%), specificity (81%), PPV (84%) and NPV (75%) of LEPs based on a disease threshold of age-adjusted normative mean values, using IENFD as the reference standard [140]. The diagnostic efficiency of evoked potentials is comparable to IENFD (Table A1) but they are non-invasive [140].

However, similar to QST, evoked potentials cannot delineate peripheral versus central nociceptive pathway pathology [129]. The limited availability of equipment and specialist nature of evoked potential evaluation limits this test to specialist neurological centres.

### 2.5. Microneurography

Microneurography detects compound action potentials of peripheral nerves that can be recorded using a Tungsten needle electrode percutaneously inserted in a nerve fascicle [136,152,153]. The electrode can be carefully manipulated to record multiple or singular action potentials from large myelinated fibres, unmyelinated fibres, mechano-sensitive C-fibres and heat-sensitive C-fibres [136,154,155,156,157]. Microelectrodes are typically inserted superficially to assess the cutaneous branches of the peroneal nerve at the fibular head [156]. The skin is stimulated with needle electrodes to identify C-fibres with comparatively slower conduction velocities of <2 ms^−1^ [153]. Thus, the action potentials during electrical stimulation are visualised in real time using a spike raster plot [157].

Microneurography has demonstrated abnormal sensitivity to thermal and mechanical stimuli which evoked a doubling of C-fibre action potentials and spiking in patients with either symptoms of neuropathy or confirmed painful neuropathy [154,155]. Further, spontaneous activity of hyper-excitable nociceptive C-fibres was identified in 87% of participants with pDPN [158]. Thus, sensitisation of C-fibres is associated with hyperalgesia in painful neuropathy [159,160]. Moreover, there are more hyper-excitable sensitised mechano-insensitive C-fibres in participants with painful compared to painless neuropathy [156]. Ørstavik et al. [157] identified a higher proportion (2:1 ratio) of mechano-insensitive to afferent mechano-responsive C-fibres in patients with pDPN or without DPN compared to controls (1:2 ratio).

However, microneurography is invasive and requires highly skilled operators. Furthermore, the protocols described for a complete recording of C-fibre function can approach 7 h per session [156] and is therefore primarily used in a research setting.

### 2.6. Current Perception Threshold

An additional modality used to measure current perception threshold is an electro-diagnostic device which assesses the functionality of Aβ, Aδ, and C type fibres by measuring current perception threshold at 2000, 250, and 5 Hz respectively [161]. Current evidence suggests it is a useful, non-invasive technique to evaluate DPN in the early, asymptomatic stages [161,162]. One study involving 558 participants with T2D, indicated that using a neurometer to measure current perception threshold identifies a greater number of DPN cases compared to monofilament testing (current perception threshold 33.9% vs. monofilament 10.6%) [163]. Similarly, a retrospective study involving 202 participants with T2D, found a greater number of subclinical DPN cases using current perception threshold compared VPT [164]. In another study involving 52 participants with T1D, observed that current perception threshold of the bilateral median and sural nerves was significantly lower in participants with diabetes [165]. Normative data from 166 healthy participants found measurements of the hand, finger and big toe are influenced by both age and sex [166]. Further studies are warranted to identify a disease cut-off value together with studies to indicate the sensitivity and specificity of current perception threshold for the screening or diagnosis of DPN.

### 2.7. NC-Stat DPN Check

The NC-Stat DPN Check is an FDA approved point-of-care nerve conduction device [167,168] that evaluates sural nerve function at the lateral malleolus [167]. Whilst there is a correlation between SNAP, SNCV and measures of small fibre function, the NC-Stat DPN Check is a measure of large fibre function and does not directly give information on small fibres [169]. In a recent study [170], there was a good correlation between standardised NCS and NC-Stat DPN Check with SNCV (*r* = 0.81) and a moderate correlation for SNAP (*r* = 0.62) [170]. The NC-Stat DPN Check has high reproducibility and can offer an objective measure of large fibre function. A intra-class correlation coefficient of 0.97 for SNAP and 0.94 for SNCV has been demonstrated with NC-Stat DPN-Check [171]. Inter-observer reproducibility compared favourably with reference NCS with correlation coefficients of 0.83 for SNAP and 0.79 for SNCV [171]. However, DPN-Check has been reported to over-estimate SNCV compared to reference NCS by a mean of +8.4 ± 6.4 m/s, representing consistent over-estimation bias [171].

In a recent cohort study of T2D participants and age and sex matched controls the diagnostic accuracy of NC-Stat DPN Check for either SNAP (<4 µV) or SNCV (<40 m/s) in one or both legs was compared with the NDS (≥3) as a clinical measure of DPN [172]. Compared to the NDS, the NC-Stat DPN Check had a sensitivity of 90.4%, specificity of 86.1%, PPV of 79.1%, NPV of 93.9%, positive likelihood ratio (LR+) of 6.51 and negative likelihood ratio (LR−) of 0.11 [172]. Smaller studies have shown that NC-Stat DPN Check has a sensitivity of 80–92% and a specificity of 80–82% for DPN [173,174,175]. The use of different disease thresholds for SNAP and SNCV in older participants and those with long-standing diabetes affects the diagnostic efficiency of NC-Stat DPN Check (Table A2). Sharma et al. [176] suggest that NC-Stat DPN Check could be used to triage and identify abnormal NCV before undertaking confirmatory NCS using standard electrophysiology equipment. However, NC-Stat DPN Check comes with a cost and any subsequent reference NCS requires specialist clinics and highly trained staff.

### 2.8. Skin Biopsy

Skin biopsy of the distal leg and thigh with quantification of intra-epidermal nerve fibre density (IENFD) is considered to be the “gold standard” to diagnose small fibre neuropathy [6,177]. The thigh and lower leg are considered to be the optimal sites for biopsy [178] to identify reduced IENFD in subjects with length-dependent neuropathy [179]. Normative age-matched data are available for IENFD [180,181]. The methods for processing skin biopsy samples and quantifying small nerve fibre pathology have been standardised and are detailed in the ENFS guidelines [177]. Specialist facilities and experience are required to produce reliable IENFD staining with PGP9.5 which is time consuming and expensive. IENFD is demonstrated using the pan-axonal marker protein gene product 9.5 (PGP-9.5), or more specifically the retrieved antigen ubiquitin carboxyl terminal hydrolase using immunohistochemistry or immunofluorescence [177,182,183,184]. IENFD has a sensitivity of 61–97% and specificity of 64–95% for identifying small fibre pathology (Table A1) [101,102,133,185,186,187,188,189,190]. IENFD cannot currently discriminate between pDPN and painless DPN [191,192]. However, deficits in regenerative capacity due to neurovascular dysfunction and inflammation may cause neuronal injury to outpace repair [46,193]. Thus, the regenerative capacity of intra-epidermal nerve fibres may help to discriminate pDPN from DPN by staining with the neuronal regeneration marker growth associated protein-43 (GAP-43) and assessing the ratio of GAP-43^+^ to PGP-9.5^+^ nerve fibres [99,194,195]. Due to contradictory published data further work is required to validate this approach [195]. IENFD was assessed in patients with T2D over 5 years and demonstrated that nerve regeneration was overtaken by neurodegeneration [196]. The rate of intra-epidermal nerve fibre loss in patients with DPN has been identified as 3.76 ± 1.46 fibres/mm per year. Although skin biopsy is minimally invasive [177], there is risk of bleeding and infection which makes this method less appealing as a screening method for DPN [197].

### 2.9. Sudoscan

Sudoscan provides a quantitative measurement of sudomotor function by quantifying electrochemical skin conductance (ESC) of the hands and feet as a measure of postganglionic sympathetic integrity [151,198]. Indeed, the EZ Scan device is an FDA approved device to identify and risk-stratify subjects with pre-diabetes or undiagnosed DM [199,200,201,202]. An abnormality in the EZ scan has been associated with progression of diabetic retinopathy and autonomic neuropathy [203,204]. Sudoscan on the other hand, is an FDA approved point of care device [205] which has been advocated as a screening tool for DPN [206]. It is quick (3–5 min) and does not require trained personnel or specialised facilities [207]. The change in conductance is calculated after stimulation of the skin by a low-voltage current (≤4 volts) through reverse iontophoresis or chronoamperometry of chloride ions [207]. Sudomotor dysfunction is more prevalent in the feet compared to the hands in people with diabetes [208].

In a study of 394 subjects with T2D, lower ESC in the feet was associated with higher NSS, NDS and VPT [209]. Notably, abnormalities in ESC have been identified in 69% of participants with asymptomatic DPN [209]. In a large cross-sectional study of 523 participants with T2D, Sudoscan was more sensitive for the detection of DPN compared to NDS and VPT with a sensitivity and specificity of 85% (AUROC = 0.88) [210]. Notably, Sudoscan in a mixed cohort of patients with distal polyneuropathy (*n* = 55; 22 with diabetes, 2 prediabetes, 31 idiopathic) and controls (*n* = 42) yielded a sensitivity of 77%, specificity 67%, PPV 59% and an NPV of 83% which was comparable to the diagnostic efficiency of IENFD of the lower leg (AUC 0.76 and 0.75 respectively) [211]. The diagnostic ability of Sudoscan in smaller cohort studies in participants with T2D are summarised in Table A3.

It has been suggested that Sudoscan may be used as an initial screening tool for DPN. However, Rajan et al. [212] have criticised the body of evidence supporting Sudoscan due to the heterogeneous normative values across different populations.

### 2.10. Neuropad

The Neuropad test kit contains two adhesive plasters containing anhydrous blue salt cobalt-II-chloride, which changes colour from blue to pink upon exposure to sweat [213] and the colour change after 10 min has been used to identify the severity of sudomotor dysfunction [214]. Neuropad allows objective assessment of sudomotor dysfunction, particularly in older patients who may lack the capacity to engage with the standard-of-care tests [215]. The simplicity and ease of interpretation of the results allows for self-assessment to identify sub-clinical DPN [216]. The time to complete colour change correlates with the MNSI [217], which correlates with DPN severity [218] and diabetes duration [219]. A significant reduction in IENFD has been demonstrated in people with diabetes and abnormal or patchy Neuropad results [220]. In a large, multi-centre, cross-sectional study 1010 participants with T2D underwent NDS as a reference test to identify DPN [219]. An abnormal Neuropad response in one or both legs was associated with a 94.9% sensitivity, 70.2% specificity, 46.3% PPV and 98.1% NPV for DPN compared with a NDS >6 [219]. Multiple cross-sectional studies have reported a high sensitivity and moderate specificity for the detection of sudomotor dysfunction (Table A4). Hewitt et al. [221] report a sensitivity of 89.4% (95% CI 83.2–93.5) and a specificity of 60.3% (95% CI 50.9–69) for the diagnosis of DPN. Moreover, sudomotor dysfunction and DPN was identified using Neuropad in 43.4% patients with recently diagnosed DM (*n* = 151) [222]. Despite the higher sensitivity the lower specificity of Neuropad compared to the 10 g monofilament [223] and lower cost-effectiveness led NICE as part of the Medical Technologies Evaluation and Diagnostics Assessment Programme [224] to not approve its use as a DPN screening test. We believe this decision is short sighted as the specificity of any test which detects disease earlier is bound to be lower.

### 2.11. Laser Doppler Flare

Cholinergic C-fibres can be activated by heating or iontophoresis of acetylcholine or histamine to induce local vasodilation [149,176,225,226,227,228,229,230,231,232,233,234,235] and the subsequent neurogenic flare can be measured using Laser Doppler flowmetry (LDF) or Laser Doppler imaging (LDI) to quantify the vasomotor axon reflex [225]. LDIFlare is significantly reduced in participants with IGT and T1D compared to controls (2.78 ± 1.1 cm^2^ vs. 5.23 ± 1.7 cm^2^; *p* < 0.0001 and 5.16 ± 2.3 cm^2^ vs. 5.23 ± 1.7 cm^2^; *p* = 0.002) [234], but does not differ between painful and painless DPN nor between participants with or without ulcers [192,236].

An LDIFlare threshold of 3.66 cm^2^ yielded a sensitivity of 75%, specificity of 85%, PPV of 74% and an NPV of 86% for the identification of DPN [230]. An age-dependent reduction of LDIFlare size of 0.56 cm^2^ per decade has been identified [231] and age-specific disease threshold values applied to the LDIFlare threshold of 3.66 cm^2^ yielded a sensitivity of 77%, specificity 90%, PPV of 82% and an NPV of 87% [230]. LDIFlare has demonstrated moderate to high sensitivity and specificity for the detection of DPN, with excellent correlation between the left and right foot (*r* = 0.95, *p* < 0.0001) [231]. At present, LDI does not have a standardised method of analysis and limited normative data and disease threshold values [225,234].

### 2.12. Corneal Confocal Microscopy

**Figure 1:**
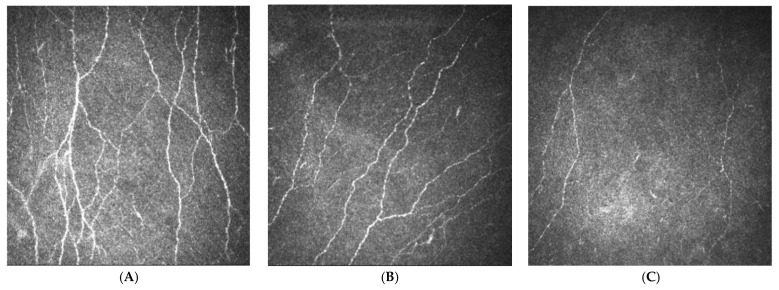
Images taken using corneal confocal microscopy in a healthy control participant (**A**), a participant with diabetes (**B**) and a participant with severe diabetic peripheral neuropathy (**C**) demonstrating the progressive corneal nerve fibre loss in the Bowman’s layer.

Over the past two decades, evaluating corneal nerve morphology has become established as a surrogate marker for diabetic and other peripheral neuropathies [237]. Corneal Confocal Microscopy (CCM) is a rapid, non-invasive, re-iterative ophthalmic imaging modality which visualises small nerve fibre in the corneal sub-basal plexus [238]. Examples of CCM images from a healthy control participant, a participant with diabetes and a participant with DPN are shown in Figure 1. CCM has demonstrated small fibre degeneration in a range of neuropathies including HIV neuropathy, idiopathic small fibre neuropathy, hereditary sensory motor neuropathy and chemotherapy-induced peripheral neuropathy [239,240,241,242,243,244,245]. Standardised corneal nerve morphometric parameters include corneal nerve fibre length (CNFL), fibre density (CNFD), branch density (CNBD), and inferior-whorl length (IWL). CCM has a high sensitivity (60–91%) and specificity (40–87%) for the diagnosis of DPN (Table A5). Decreased corneal nerve parameters have been reported in people with impaired glucose tolerance [246,247], T1D [248,249,250,251,252,253] and T2D [254,255,256,257]. Moreover, reductions in corneal nerve fibres occur in patients with clinically confirmed DPN compared to those without DPN and correlate with DPN severity [258,259,260,261,262,263,264]. The Addition-Denmark study of participants with T2D found no difference in CNFD between participants with and without DPN [86], however the corneal nerve analysis was undertaken using automated analysis and both groups had good and comparable metabolic control [86]. A recently published study identified corneal nerve loss in participants with T1D and T2D compared to controls with good diagnostic accuracy for participants with DPN [265].

A reduction in corneal nerve measures may precede reduced corneal sensitivity in patients with diabetes [266]. In a large study of 590 patients with diabetes, rapid loss of ≥6% of CNFL per year occurred in 17% of participants and was associated with the development of DPN [267]. Thus, a rapid decline in corneal nerves may help to stratify patients at highest risk for the development and progression of DPN. A rapid decline in CNFL, CNFD and CNBD preceded the development of foot ulceration and Charcot foot whilst VPT and QST remained unchanged, suggesting CCM could identify high-risk patients [258,268]. CCM can detect early nerve regeneration with no change in other measures of neuropathy in people with T1D after simultaneous pancreas and kidney transplantation [14,16,269].

Normative reference values have been reported from 343 healthy volunteers with a linear age-dependent decrease in CNFL (−0.045 mm/mm^2^; *p* = 0.07 and −0.060 mm/mm^2^; *p* = 0.02) and CNFD (−0.164 no/mm^2^; *p* < 0.01 and −0.161 no/mm^2^; *p* < 0.01) in both men and women [270]. A pooled multi-national consortium study of 998 participants with T1D (*n* = 516) and T2D (*n* = 482) has demonstrated that a disease threshold of <8.6 mm/mm^2^ can be used to diagnose DPN, whilst a threshold ≥15.3 mm/mm^2^ is sufficient to exclude DPN using fully automated analysis with an equal error rate of 88% specificity and 88% sensitivity [271]. Automated analysis is much quicker than manual human annotation with comparable performance for the detection of DPN [261]. Notably, automated analysis of nerve fibres in CCM images is capable of distinguishing between patients with and without DPN minimising inter/intra observer variability [272,273,274]. The application of a deep learning algorithm on CCM images from 90 healthy participants and 132 people with DPN had a specificity of 87% and sensitivity of 68% for the identification of DPN [275]. 

CCM is widely used in specialist ophthalmology centres worldwide and has a growing user base in neurology which has increased from an initial 3 centres to over 100 centres worldwide within the last two decades. CCM maytherefore be deployed in a screening programme alongside diabetic retinal screening to monitor the development and progression of DPN [276]. Future upscaling in the general clinical (non-research) arena requires the mitigation of barriers for adoption into clinical practice. General clinician acceptance and cost-effectiveness models are still required to tackle downstream barriers. A future plan for clinical adoption will need to tackle both internal (consumer awareness and engagement with CCM manufacturers) and internal factors (structures to encourage adoption, trials/studies of efficacy and policy). A robust framework for generating evidence for deploying this diagnostic methodology will require adoption [2]. However, given the rapid expansion of CCM as a tool for diabetic neuropathy research, additionally with recent clinical trials utilising it as a surrogate endpoint [1], there is a clear expectation for the continued increased use in CCM.

### 2.13. Limitations of This Review

The primary limitation of this critical appraisal is that no formal tool for the assessment of bias or methodological quality was performed. Therefore, the appraisal of the included articles is limited to the authors’ opinions without the utilisation of a validated critical appraisal tool. Additionally, a peer reviewed search strategy and protocol were not published prior to the submission of this manuscript. Significant heterogeneity existed between the studies including different clinical settings, screening tools and diagnostic methods as comparators for the detection of DPN, thus resulting in a challenging comparative interpretation of the diagnostic efficacy of each method. Notably, the majority of articles include participants with a longer duration of diabetes. Any future systematic reviews of the diagnostic efficacy should formally account for study heterogeneity, e.g., study methodology, participant’s characteristics, and definition of DPN.

## 3. Discussion

**Figure 2:**
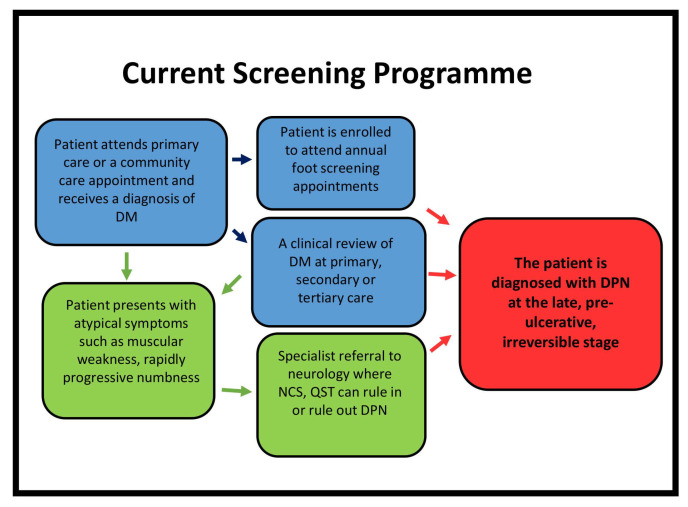
Clinical pathway for patients diagnosed with diabetes mellitus. DM—diabetes mellitus; DPN—diabetic peripheral neuropathy; NCS—nerve conduction studies; QST—quantitative sensory testing.

The identification of early DPN allows for proactive multi-factorial intervention to limit progression of nerve damage. Although guidance exists for the use of a range of simple diagnostic modalities [6,78], current screening techniques detect advanced disease, where interventions are ineffective as summarized in Figure 2. NCS together with signs and symptoms are not sensitive for identifying subclinical DPN. QST is subjective and cannot differentiate between central and peripheral nerve damage. The DNFS static QST protocol identifies patterns of small fibre deficits and may be key to assessing optimal therapeutic response. However, the application of the full battery of tests is time-consuming and requires training. LDIFlare is non-invasive, relatively fast and is associated with a high sensitivity and specificity for DPN. However, there are no published disease threshold values and no standardized method of image analysis. Skin biopsy is considered to be the reference standard for the identification of small fibre neuropathy. However, mass screening and repeat biopsies are not feasible. LEPs, CHEPs, Sudoscan and Neuropad are alternatives but lack robust data regarding diagnostic and prognostic ability. The use of screening tests to identify small fibre pathology in people with diabetes alongside retinopathy screening in a one stop microvascular screening appointment was recently implemented enabling new diagnoses of DPN, which was valued by patients [277].

**Figure 3:**
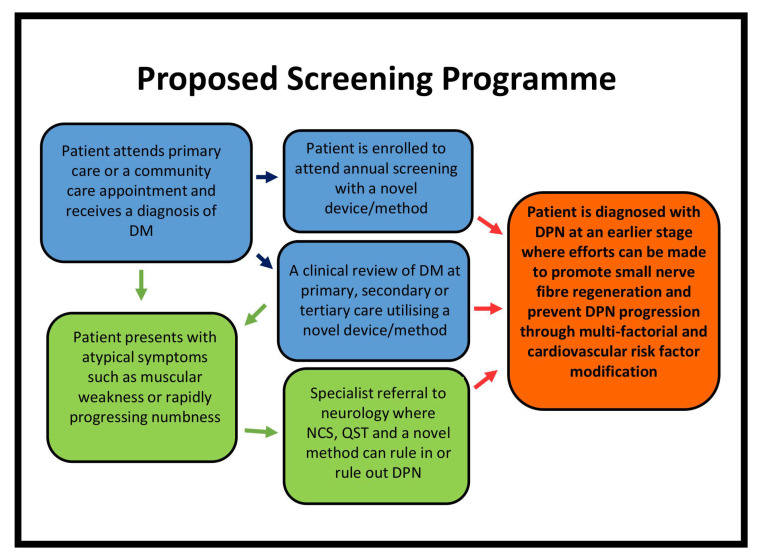
Proposed clinical pathway for patients diagnosed with diabetes mellitus using a new screening method. DM—diabetes mellitus; DPN—diabetic peripheral neuropathy; NCS—nerve conduction studies; QST—quantitative sensory.

As such, a new methodology to detect early changes in DPN should be implemented at key stages of patient assessment as shown in Figure 3. CCM provides detailed quantification of small nerve fibres and predicts the development and progression of DPN. The anatomically distinct area of examination (cornea) from the perceived primary area of pathology (feet and hands) have been debated as a possible limitation of CCM. However, as the need for corneal transparency decrees the lack of vasculature, and unlike unmyelinated fibres elsewhere, corneal nerve fibres have a significant vulnerability to degeneration from metabolic, toxic, immune or inflammatory insults [1]. Corneal nerve fibre pathology is demonstrated in a number of systemic and central neurodegenerative diseases, e.g., Parkinson’s, multiple sclerosis, stroke [3,4]; this is suggestive that structural changes in corneal nerves are not entirely specific to the peripheral nervous system [5]. Clearly, the corneal sub-basal nerve plexus is vulnerable to a number of disease mechanisms and corneal nerve pathology may manifest in diverse conditions. Whereas, DPN is characterised by a distal and symmetric degeneration of sensory nerves. CCM conveniently images exactly these most distal sensory nerves of the cornea. There is now a robust evidence base for the role of CCM in DPN. Importantly, recent studies identifying greater corneal pathology of the inferior whorl (a vertex of nerves located infero-nasally) may discriminate between pDPN and insensate DPN [6,7]. The prognostic role of the inferior whorl in those with lower inferior whorl nerve length in the development of neuropathic pain requires further investigation. The early detection of DPN is essential in the prevention of long-term sequelae and in reducing morbidity. Early detection of DPN requires the assessment of small nerve fibres, which are of paramount importance. CCM is rapid, non-invasive and readily repeatable, providing objective, reproducible and sensitive quantification of small nerve fibres and the detection of nerve degeneration and regeneration. CCM is a game changer in the diagnosis and evaluation of DPN and represents the most pertinent modality for early detection.

## Appendix A

**Table A1:** Studies of the sensitivity and specificity of skin biopsy for the diagnosis of DPN.

Citation	Participants	Comparator Test (s)	Cut Disease off Values Used	Threshold Value Used on Index Test	Test	AUC	Sensitivity %	Specificity %	PPV	NPV	+LR	−LR
McArthur et al., 1998 [190]	Control = 98 Sensory Neuropathy = 20	No Comparator Test	NA	>the 5th percentile of the normative data	IENFD Distal Leg	-	45	97	92	90	-	-
Chien et al., 2001 [179]	Control = 55 Idiopathic = 28 DM = 5 Vasculitis = 2	NCS	Reduced amplitudes of sensory action potential in the median, ulnar and sural nerves	>the 5th percentile of the normative data	IENFD Distal Forearm	-	70	94	84	90	-	-
		HDT	-									
		CDT	-		IENFD Distal Leg	-	80	95	84	93	-	-
		Neurological Examination	-									
Koskinen et al., 2005 [189]	DPN = 12 Idiopathic/CIPN /Amyloidosis = 10 CIDP = 7 Control = 15	Neurological Examination	-	-	NPEA Distal Leg	-	90	95	95	91	-	-
		VPT	-									
		CDT	-									
		NCS	-									
Vlcková-Moravcová et al., 2008 [185]	SFN = 58 MFN = 41 Control = 37	NDS	(none = 0–2, mild = 3–5, moderate 6–8, severe 9–10)	≤8.80/mm	IENFD All neuropathy participants	0.89	79.7	82.1	-	-	-	-
		NSS	≥1	≤7.77/mm	IENFD SFN Group	0.88	68.6	95	-	-	-	-
		MNSI	≥2	≤8.80/mm	IENFD MFN Group	0.87	72.2	79.6	-	-	-	-
		CDT	CDT—see below *									
		HDT	WDT—see below *									
		Touch	-									
		Proprioception	-									
		Vibration	-									
		NCS	Abnormal conduction study from at least two nerves									
Devigili et al., 2008 [133]	LFN = 22 SFN = 67 MFN = 21 Total = 110	Thermal Thresholds	>95th percentile values of participants compared to age and sex matched controls considered abnormal	7.63/mm	IENFD Total Distal Leg	0.90	82.8	90	89.4	87.5	-	-
		NCS	SNAP <20 mv and <50 m/s (fifth to second finger) SNAP <6 and >42 m/s (sural) CMAP—A difference of at least 50% in SNAP and CMAP amplitude to define significant asymmetry (peroneal, tibial, ulnar and median)	12.8/mm	IENFD Total Proximal Thigh	0.82	95.5	57.5	-	-	-	-
		Laser Doppler Flowmetry	-	>95th percentile values of participants compared to age and sex matched controls considered abnormal	CDT and HDT	0.57	56.7	36.5	48.7	44.2	-	-
		LEPs	Lower intensity compared to 18 age and sex matched controls									
Nebuchennykh et al., 2009 [102]	SFN = 45 MFN = 165 Total = 210	NISLL	-	10.3 fibres/mm	Distal IENFD—SFN	0.75	78	64	-	-	-	-
		NCS	>2 SD compared to reference normal values in ≥2 nerves	9.8 fibres/mm	Distal IENFD MFN and SFN	0.77	73	69	-	-	-	-
		QST	For reference values see [182]									
Alam et al., 2017 [101]	Control = 27 T1D without DPN = 30 T1D with DPN = 31	NSP	-	<4.5 fibres/mm	T1D with DPN	0.73	61	80	-	-	-	-
		McGill-SF-2										
		CDT	≤24.7 °C									
		HDT	≥38.0 °C									
		VPT	≥13 V									

AUC—area under the curve; CARTs—cardiac autonomic reflex tests; DM—Diabetes Mellitus; DNI—diabetic neuropathy index; DPN—diabetic peripheral neuropathy; IENFD—intra-epithelial nerve fibre density; MFN—Mixed-Fibre Neuropathy; MNSI—Michigan neuropathy screening instrument; NCS—nerve conduction studies; NDS—neuropathy disability score; NISLL—neuropathy impairment score of the lower limbs; NPV—negative predictive value; NSS—neuropathy symptom score; NTSS—neuropathy total symptom score; PPV—positive predictive value; QAFT—quantitative autonomic function testing; QSART—quantitative sudomotor axon reflex test; SFN—Small Fibre Neuropathy; TCNS—Toronto Clinical Neuropathy Score; T1D—Type 1 diabetes; T2D—Type 2 diabetes; TDT—thermal detection thresholds; UENS—Utah Early Neuropathy Scale; VPT—vibration perception threshold. * Men Aged 20–40 = <26.3 °C; Men Aged 40–60 = <25.5 °C; Men Aged 60+ = <22.8 °C; Women Aged 20–40 = <29.1 °C; Women Aged 40–60 = <26.6 °C; Women Aged 60+ = <21.1 °C HDT—Men Aged 20–40 = >40.8 °C; Men Aged 40–60 = >44.9 °C; Men Aged 60+ = <46.2 °C; Women Aged 20–40 = >39.5 °C; Women Aged 40–60 = >41.2 °C; Women Aged 60+ = >48.2 °C

**Table A2:** Studies of sensitivity and specificity of NC-Stat DPN Check for the diagnosis of DPN.

Citation	Participants	Comparator Test (s)	Cut off Value	Threshold Value Used on Index Test	Test and Target Condition	AUC	Sensitivity (%)	Specificity (%)	PPV	NPV	+LR	−LR
Chatzikosma et al., 2016 [172]	T2D = 114 Controls = 46	NDS	≥3	Abnormality of ≥1 in ≥1 leg	Sural SNAP/SNCV—DPN	-	90.4	86.1	79.1	93.9	6.51	0.11
Perkins et al., 2006 [174]	T1D = 8 T2D = 64	Reference NCS	≥1 sign or symptom + abnormality of at least two parameters in at least two nerves [83]	Abnormality in either SNCV or SNAP	Sural SNAP/SNCV—DPN	-	92	82	92	82	-	-
Kural et al., 2019 [277]	T2D (*n* = 168)	Reference NCS	DPN ruled in if the sum *z*-score >2 [278]	<6 µV Both Legs	Sural SNAP—DPN	-	96	71	54	98	-	-
				>4 and ≤6 µV Both Legs	Sural SNAP—DPN	-	95	84	68	98	-	-
Perkins et al., 2008 [173]	DM = 72	Reference NCS; PMNAP, PMNCV, tibial SNCV, tibial SNAP, median SNAP, median SNCV	≥1 sign or symptom + abnormality of at least two parameters in at least two nerves [83]	Abnormality in either SNCV or SNAP	Sural SNAP—DPN	-	88	82	-	-	-	-
Scarr et al., 2018 [175]	T1D = 68 Controls = 71	Reference NCS; SNAP SNCV	≤7.2 µV for participants ≤ 65 years old, ≤5.5 µV for participants ≥ 65 years old ≤40 m/s	Abnormality in either SNCV (≤44 m/s) or SNAP (<6 µV) or both measures	SNAP only SNCV only SNAP/SNCV SNAP and SNCV	- - - 0.88	80 81 86 66	80 82 79 97	- - - -	- - - -	- * - * - * - *	- - - -

AUC—area under the curve; CARTs—cardiac autonomic reflex tests; CNBD—corneal nerve fibre branch density; CNFD—corneal nerve fibre density; CNFL—corneal nerve fibre length; CNFT—corneal nerve fibre tortuosity; DAN—diabetic autonomic neuropathy; DM—Diabetes Mellitus; DPN—diabetic peripheral neuropathy; IENFD—intra-epithelial nerve fibre density; LR+—positive likelihood ratio; LR−—negative likelihood ratio; MNSI—Michigan neuropathy screening instrument; NCCA—noncontact corneal aesthesiometer; NCS—nerve conduction studies; NDS—neuropathy disability score; NISLL—neuropathy impairment score of the lower limbs; NPV—negative predictive value NTSS—neuropathy total symptom score; PMNAP—peroneal motor nerve amplitude; PMNCV—peroneal motor nerve conduction velocity; PPV—positive predictive value; QAFT—quantitative autonomic function testing; QSART—quantitative sudomotor axon reflex test; SNAP—sensory nerve action potential; SNCV—sensory nerve conduction velocity; T1D—Type 1 diabetes; T2D—Type 2 diabetes; TCNS—Toronto Clinical Neuropathy Score; TDT—thermal detection thresholds; UENS—Utah Early Neuropathy Scale; VPT—vibration perception threshold. * Positive likelihood ratios are stated in the text but are not for optimal thresholds as indicated by the receiver operating characteristic area under the curve.

**Table A3:** Studies of the sensitivity and specificity of Sudoscan for the detection of DPN.

Citation	Participants	Comparator Test (s)	Cut Disease off Values Used	Threshold Value Used on Index Test	Test and Target Condition	AUC	Sensitivity (%)	Specificity (%)	PPV	NPV	+LR	−LR
Krieger et al., 2018 [279]	T2D = 47 DPN =27 Non-DPN = 20 Controls = 16	NDS	Mild (3–4) Moderate (5–6) Severe (7–10)	ESC of ≤80.00 μS Foot	Sudoscan—DPN	0.70	70	53	-	-	-	-
		NDS		ESC of ≤75.00 μS Hand	Sudoscan—DPN	0.71	85	50	-	-	-	-
Goel et al., 2017 [210]	Total (T2D) = 523 DPN = 110 Non-DPN = 413	NDS	DPN = ≥6 Non-DPN = <6	Mean ESC <60 μS	Sudoscan—DPN	0.88	85	85	-	-	-	-
					VPT—DPN	0.84	72	90	-	-	-	-
		VPT	>15 considered abnormal		NDS—DPN	0.6	52	60	-	-	-	-
Smith et al., 2014 [211]	Total = 97 Suspected DSP = 55 Control = 42	UENS	-	≤ 70 μS	Sudoscan—DPN	0.76	77	67	59	83	-	-
		IENFD	-									
		QSART	-	-	IENFD—DPN	0.75	63	63	73	52	-	-
		NCS	-									
Selvarajah et al., 2015 [208]	Total = 70 T1D = 45 Control = 25	NTSS NISLL TDT VPT NCS CARTS	>1 Symptom/sign and abnormal NCS	≤ 77.0 μS	Sudoscan DPN	0.85	87.5	76.2	-	-	-	-
			Autonomic Function Score	>30.0% Cardiac Autonomic Neuropathy Risk Score	Sudoscan CAN	0.75	65	80	-	-	-	-
Yajnik et al., 2012 [280]	T2D = 265	MNSI VPT Ewing’s CART	>2 >21 V HRV E/I Ratio <1.21 HRV 30/15 Ratio <1.03 HRV during Valsalva manoeuvre <1.20 Orthostatic BP response >20 mmHg	<52.0 μS	Sudoscan DPN or CAN	0.71	73	62	-	-	-	-
Casellini et al., 2013 [207]	T1D = 83 Control = 210	Modified NISLL HDT CDT QAFT	- ≥12.4 °C ≥10.5 °C -	>60 µS = Normal 60–40 µS = Moderate <40 µS = Severe	Sudoscan Hand—DPN	0.86	78.3	85.7	61	93.2	5.48	0.25
					Sudoscan Feet—DPN	0.88	78.3	92.3	74.6	93.72	10.28	0.23
					Modified NISLL	0.84	76.6	85.7	95.8	46.1	5.37	0.27
Carbajal-Ramírez et al., 2019 [281]	Total T2D = 221 T2D <5 years = 170 T2D ≥5 years = 51	MNSI VPT 10-g Monofilament	- - -	<60 μS = abnormal hands AND/OR <70 μS = abnormal feet	Sudoscan T2D <5 years Sudoscan T2D ≥5 years	0.66 0.72	91 97	- -	88 87	- -	- -	- -
Binns-Hall et al., 2018 [282]	Total = 236 (T2D = 97.8%) No DPN = 163 Mild DPN = 34 Moderate DPN = 19 Severe DPN = 20	TCNS 10-g Monofilament NCS (DPN Check)	TCNS- >5 Inability to feel ≥2 sites taken to indicate DPN If either SNAP was >4 µV and/or SNCV was <40 m/s	≤58.5 µS (>60 µS = Normal <60 µS = DPN) ≤4.3 ≤46.3	Sudoscan— DPN	0.75	77.4	68.3	-	-	-	-
					NC-Stat DPN Check SNAP	0.84	84.3	72.3	-	-	-	-
					NC-Stat DPN Check SNCV	0.81	68.3	80	-	-	-	-
					10-g Monofilament DPN	0.61	30	92.7	-	-	-	-
					Sudoscan + NC-Stat DPN Check	0.73	93.2	52.8	-	-	-	-
Eranki et al., 2013 * [283]	T2D = 309	VPT	- VPT ≥ 15		Sudoscan VPT	0.68 0.65	82 82	61 55	- -	- -	- -	- -

AUC—area under the curve; CARTs—cardiac autonomic reflex tests; DPN—diabetic peripheral neuropathy; DSP—distal symmetric polyneuropathy; IENFD—intra-epithelial nerve fibre density; MNSI—Michigan neuropathy screening instrument; NCS—nerve conduction studies; NDS—Neuropathy Disability Score; NISLL—neuropathy impairment score of the lower limbs; NPV—negative predictive value NTSS—neuropathy total symptom score; PPV—positive predictive value; QAFT—quantitative autonomic function testing; QSART—quantitative sudomotor axon reflex test; T1D—Type 1 diabetes mellitus; T2D—Type 2 diabetes mellitus; TCNS—Toronto Clinical Neuropathy Score; TDT—thermal detection thresholds; UENS—Utah Early Neuropathy Scale; VPT—vibration perception threshold. All sensitivities and specificities for Sudoscan are of the feet to detect DPN unless otherwise stated. * Detection of microvascular complications and autonomic risk.

**Table A4:** Studies of the diagnostic ability of Neuropad for the detection of abnormal sudomotor function, cardiac autonomic neuropathy and DPN.

Citation	Participants	Comparator Test (s)	Cut Disease off Values Used	Threshold Value Used on Index Test	Test and Target Condition	AUC	Sensitivity (%)	Specificity (%)	PPV	NPV	+LR	−LR
Papanas et al., 2011 [218]	T2D = 251	NDS	Mild ≥ 3	<365 s	Neuropad DPN	0.91	95	75	99	21	3.79	0.07
		NDS	Moderate ≥ 6	≤1005 s	Neuropad DPN	0.96	91	96	96	92	24.4	0.09
		NDS	Severe ≥ 9	≥1190 s	Neuropad DPN	0.97	91	95	84	98	19	0.09
Papanas et al., 2005 [213]	T2D = 104	DNI	Moderate = 2.5–4.5	Normal—A complete colour change (blue to pink) in both feet after 10 min.	Neuropad DPN	-	94.4	69.7	-	-	-	-
			Severe = 5–8	Abnormal—Partial or incomplete colour change in one foot after 10 min.								
Liatis et al., 2007 [284]	Total = 117 T1D = 9 T2D = 108	NSS	≥3	Normal—A complete colour change (blue to pink) in both feet after 10 min. Abnormal—Partial or incomplete colour change in one foot after 10 min.	Neuropad DPN	-	86	67.2	66.2	86.5	-	-
		Ewing’s Score	≥2		Neuropad CAN	-	59.1	46.5	40.6	64.7	-	-
		Ewing’s Score	≥6		Neuropad CAN	-	80.9	50	26.0	92.1	-	-
Papanas et al., 2007 [217]	T2D = 120	MNSI	0—none 1—mild 2—moderate 3—severe neuropathy	≤530 s	Neuropad DPN	-	97	100	-	-	-	-
				≤1000 s	Neuropad DPN	-	100	97	-	-	-	-
				<1440 s	Neuropad DPN	-	93	100	-	-	-	-
				>1440 s	Neuropad DPN	-	100	99	-	-	-	-
Papanas et al., 2008 [285]	T2D = 154	NDS		Abnormal >25 V Intermediate 15–25 V Normal <15 V	Neuropad—DPN	-	97.8	67.2	-	-	-	-
					VPT—DPN	-	78.9	85.9	-	-	-	-
Quattrini et al., 2008 [220]	Total n = 57 T1D = 20 T2D = 37	NDS	≥3	Partial or incomplete colour change in one foot after 10 min	Neuropad— DPN	-	85	45	69	71	-	-
Tentolouris et al., 2008 [216]	Total = 156 T1D = 7 T2D = 149	NSS		Normal (pink colour bi-laterally) or abnormal (blue colour or any other combinations of colours bilaterally)	Neuropad— DPN	-	87	66	94	79	-	-
Bilen et al., 2007 [286]	T2D = 105	Corrected QT Interval	>0.440 s	Blue indicated sudomotor dysfunction and pink indicated normal sudomotor function	Neuropad— CAN	-	87.5	43.1	48.6	84.8	-	-
Freitas et al., 2009 [287]	T2D = 40 DPN+ = 22 DPN− = 18	NDS	≥6	Absence of a colour change from blue to pink, or incomplete change (blue and pink mixed) were considered to correspond to an altered test	Neuropad— DPN	-	100	44	69	100	-	-
Ishibashi et al., 2014 [288]	Control = 28 T2D = 78 DPN Stage 1 = 23 DPN Stage 2 = 28 DPN Stage 3 = 20 DPN Stage 4 = 7	1. Subjective symptom in bilateral lower limbs or feet 2. Loss of or decreased ankle jerk 3. Decreased VPT assessed	≥2	>815 s	Neuropad— DPN	-	83.1	84.0	-	-	-	-
Manes et al., 2014 [219]	Total T2D = 1010 Sudomotor Dysfunction = 441 No Sudomotor Dysfunction = 569	NDS	>6	Partial colour change = 1 No colour change = 2 Blue to pink = 0 Neuropad score ≥ 1 Neuropad score = 2	Neuropad Sudomotor Dysfunction	-	85.6	71.2	51.2	93.3	-	-
					Neuropad DPN	-	94.9	70.2	46.3	98.1	-	-
					Neuropad Sudomotor Dysfunction	-	52	96	82	85	-	-
					Neuropad DPN	-	64	96	82	91	-	-
Kamenov et al., 2010 [289]	Total = 264 T1D = 61 T2D = 203	NDS NDS	>3 >6		Neuropad Neuropad	- -	76.3 79.3	56.1 42.9	86.3 62.8	39.5 63.0	- -	- -
Ponirakis et al., 2014 [290]	T2D = 50	DNS	>2	Scanned images of Neuropads applied (*n* = 50) were estimated by three masked observers on two separate occasions on a scale of 0-100% colour change	Neuropad *	66	70	50	63	57	1.4	0.6
		VPT	>14 volts		Neuropad *	73	83	53	45	39	1.77	0.32
		SNAP	<3 µV		Neuropad *	70	70	64	26	92	1.94	0.47
		SNCV	<43 m/s		Neuropad *	63	64	54	45	72	1.39	0.67
		PMNAP	<2 mV		Neuropad *	69	82	50	31	91	1.64	0.36
		PMNCV	<42 m/s		Neuropad *	70	81	54	59	78	1.76	0.35
		WPT	>43 °C		Neuropad *	60	68	49	26	44	1.33	0.65
		CNFL	<24 no/mm^2^		Neuropad *	67	74	60	54	78	1.85	0.43
		CNFD	<14 mm/mm^2^		Neuropad *	85	83	80	49	95	4.12	0.21
		NSS	(Subjective Test)		Neuropad *	68	78	60	34	91	1.95	0.37
Ziegler et al., 2011 [222]	T1D = 52	NDS	≥2	Normal—A complete colour change (blue to pink) in both feet after <10 min. Abnormal—Partial or incomplete colour change in one foot after 10, 15, or 20 min	Neuropad—DPN 10 min	-	87.5	47.7	23.3	95.4	-	-
					Neuropad— DPN 15 min	-	50	70.4	23.5	88.6	-	-
					Neuropad— DPN 20 min	-	12.5	84.1	12.5	84.1	-	-
					Neuropad *— 10 min	-	80	44.7	13.3	95.4	-	-
					Neuropad *— 15 min	-	80	72.3	23.5	97.1	-	-
					Neuropad *— 20 min	-	20	85.1	12.5	90.9	-	-
Ziegler et al., 2011 [222]	T2D = 99	NDS	≥2	Normal—A complete colour change (blue to pink) in both feet after <10 min. Abnormal—Partial or incomplete colour change in one foot after 10, 15, or 20 min	Neuropad— DPN 10 min	-	65.1	48.2	49.1	64.3	-	-
					Neuropad— DPN 15 min	-	48.8	76.8	61.8	66.1	-	-
					Neuropad— DPN 20 min	-	34.9	89.3	71.4	64.1	-	-
					Neuropad *— 10 min	-	67.7	47.1	36.8	76.2	-	-
					Neuropad *— 15 min	-	45.2	70.6	41.2	73.8	-	-
					Neuropad *— 20 min	-	32.3	83.8	47.6	73.1	-	-

AUC—area under the curve; CARTs—cardiac autonomic reflex tests; DNI—diabetic neuropathy index; DPN—diabetic peripheral neuropathy; IENFD—intra-epithelial nerve fibre density; LR+—positive likelihood ratio; LR−—negative likelihood ratio; MNSI—Michigan neuropathy screening instrument; NCS—nerve conduction studies; NDS—Neuropathy Disability Score; NISLL—neuropathy impairment score of the lower limbs; NPV—negative predictive value; NSS—neuropathy symptom score; NTSS—neuropathy total symptom score; PMNAP—peroneal motor nerve amplitude; PMNCV—peroneal motor nerve conduction velocity; PPV—positive predictive value; QAFT—quantitative autonomic function testing; QSART—quantitative sudomotor axon reflex test; T1D—type 1 diabetes; T2D—type 2 diabetes; TCNS—Toronto Clinical Neuropathy Score; TDT—thermal detection thresholds; UENS—Utah Early Neuropathy Scale; VPT—vibration perception threshold. * For the detection of sudomotor dysfunction.

**Table A5:** Studies of the sensitivity and specificity of corneal confocal microscopy for the diagnosis of DPN.

Citation	Participants	Comparator Test (s)	Cut-off Value *	Index Test Threshold	Test and Target Condition	AUC	Sensitivity	Specificity	PPV	NPV	+LR	−LR
Ahmed et al., 2012 [291]	T1D = 89 Control = 64	TCNS LDIFlare	- -	≥11.50 mm/mm^2^	CNFL—DPN	-	-	93	83	-	8.5	-
				≥14.00 mm/mm^2^	CNFL—DPN	0.88	85	84	76	90	5.3	0.18
				≥15.80 mm/mm^2^	CNFL—DPN	-	91	-	-	91	-	0.16
				-	CNFD	0.84	-	-	-	-	-	-
				-	CNBD	0.73	-	-	-	-	-	-
Perkins et al., 2018 [271]	Total = 998 T1D = 516 T2D = 482	Reference standard based on recommendations [78]	Abnormal nerve conduction and signs or symptoms of neuropathy [6]	12.5 mm/mm^2^	Automated CNFL T1D—DPN	0.77	73	69	50	86	2.32	0.39
				12.3 mm/mm^2^	Automated CNFL T2D—DPN	0.68	69	63	66	66	1.86	0.49
				12.3 mm/mm^2^	Automated CNFL T1D and T2D—DPN	0.77	67	66	59	74	1.97	0.50
				Total <8.6 mm/mm^2^	Automated CNFL T1D and T2D—DPN	-	88	88	-	-	-	-
Alam et al., 2017 [101]	T1D with neuropathy = 31 Control Participants = 27	CDT HDT VPT NCS Autonomic Function Tests IENFD	≤24.7 °C ≥38.0 °C ≥13 V	25 no/mm^2^ 36.5 no/mm^2^ 16.8 mm/mm^2^ <4.5 no/mm	CNFD—DPN CNBD—DPN CNFL—DPN IENFD	0.81 0.67 0.74 0.73	77 58 61 61	79 79 86 80	- - - -	- - - -	- - - -	- - - -
Chen et al., 2015 [292]	T1D = 63 Control = 26	DNS NDS CDT HDT VPT NCT (PMNamp)	≥1 ≥3 NS NS NS PMNCV of <42 m/s	2 SD below the mean of the control group	Manual CNFD—DPN CNFL—DPN CNBD—DPN Automated CNFD—DPN CNFL—DPN CNBD—DPN IENFD	0.82 0.70 0.59 0.80 0.77 0.80 0.66	82 59 17 60 59 29 53	71 74 96 83 80 98 76	- - - - - - -	- - - - - - -	- - - - - - -	- - - - - - -
Edwards et al., 2014 [293]		NDS 10-g Monofilament NCS	Abnormal nerve conduction and signs or symptoms of neuropathy	-	CNFL	0.64	32	87	-	-	-	-
				-	Tortuosity-standardised CNFL	0.67	38	88	-	-	-	-
Ishibashi et al., 2012 [251]	T1D = 38 Control = 38	Signs and symptoms VPT Reflex Testing	≥2 of the following: bilateral lower limb symptoms, impaired reflexes and abnormal VPT	11 mm/mm^2^	CNFL	-	87	77	-	-	-	-
				28.4 mm^2^	CNFD	-	87	76	-	-	-	-
				25.2/0.1 mm	CNFT Grade	-	92	94	-	-	-	-
				2.47	Beading Frequency	-	81	79	-	-	-	-
Ponirakis et al., 2015 [294]	DPN− = 64 DPN+ = 46	NDS PMNCV Signs and Symptoms	>2 >42 μV ≥1	<14 mm/mm^2^ <18 no/mm^2^ <24 no/mm^2^ <4 no./mm >42 °C >2 >14 <3 μV <43 μV <2 μV <42 μV	CNFL CNFD CNBD IENFD HDT NDS VPT Sural SNAP Sural SNCV PMNAP PMNCV	0.80 0.82 0.79 0.63 0.69 0.67 0.75 0.86 0.62 0.63 0.60	89 88 83 65 75 71 80 85 66 67 62	75 78 72 54 60 58 71 83 61 54 58	- - - - - - - - - - -	- - - - - - - - - - -	- - - - - - - - - - -	- - - - - - - - - - -
Tavakoli et al., 2015 [295]	DAN+ve = 19 DAN−ve = 15 Control = 18	CASS	>2	<4.78 mm/mm^2^ <23.26 no/mm^2^ <19.53 no/mm^2^	CNFL—DAN CNFD—DAN CNBD—DAN	0.90 0.91 0.88	86 86 100	78 78 56	- - -	- - -	- - -	- - -
Pritchard et al., 2015 [296]	Control = 80 T1D = 107 of which; DPN+ = 25 DPN− = 82	NDS *	≥3 ≥3	≤17.9 ≤18.6	CNFL Centre CNFL Whorl	0.76 0.77	90 80	50 60	- -	- -	2.0 2.2	0.2 0.3
Wang et al., 2020 [297]	Total = 220 Control = 48 T2D = 172	Signs and Symptoms PMNCV SSNCV SSNAMP PCMAP	≥42 m/s ≥42 m/s ≥6 µV ≥2 mV	<15.3 n/mm^2^ <39 n/mm^2^ <25.68 n/mm^2^	CNFL CNBD CNFD	0.70 0.67 0.66	80 85 78	59 47 52	- - -	- - -	1.98 1.61 1.65	0.33 0.31 0.41
Petropoulos et al., 2014 ** [261]	T1/2D = 186 DPN+ = 100 DPN− = 86 Controls = 55	PMNAP Sural SNAP PMNCV HDT	<1.4 µV <5.5 µV <42.0 m/s >41 °C	15.8 mm/mm^2^ 18.7 no/mm^2^ 41.7 no/mm^2^ 14.6 mm/mm^2^ 14.9 no/mm^2^ 14.9 no/mm^2^ 19.4 mm/mm^2^ 23.1 no/mm^2^ 47.1 no/mm^2^ 16.1 mm/mm^2^ 18.9 no/mm^2^ 23.4 no/mm^2^ 19.7 mm/mm^2^ 25.4 no/mm^2^ 49.0 no/mm^2^ 16.0 mm/mm^2^ 19.7 no/mm^2^ 24.9 no/mm^2^ 19.2mm/mm^2^ 24.0 no/mm^2^ 47.2 no/mm^2^ 15.9 mm/mm^2^ 17.9 no/mm^2^ 22.9 no/mm^2^	CNFL Manual CNFD Manual CNBD Manual CNFL Auto CNFD Auto CNBD Auto CNFL Manual CNFD Manual CNBD Manual CNFL Auto CNFD Auto CNBD Auto CNFL Manual CNFD Manual CNBD Manual CNFL Auto CNFD Auto CNBD Auto CNFL Manual CNFD Manual CNBD Manual CNFL Auto CNFD Auto CNBD Auto	0.82 0.84 0.75 0.84 0.84 0.79 0.70 0.74 0.65 0.77 0.72 0.70 0.73 0.74 0.68 0.79 0.74 0.67 0.67 0.69 0.65 0.68 0.67 0.64	77 79 73 77 76 74 68 72 61 72 18.9 23.4 74 78 69 74 80 68 63 63 65 61 63 60	76 78 68 74 72 73 67 67 56 66 56 54 63 70 61 71 61 52 61 62 55 61 60 58	- - - - - - - - - - - - - - - - - - - - - - - -	- - - - - - - - - - - - - - - - - - - - - - - -	3.2 4.6 2.3 3.3 3.4 2.9 2.1 1.9 1.4 2.1 1.9 1.4 2.0 2.6 1.8 2.6 2.2 1.4 1.6 1.7 1.4 1.5 1.5 1.4	0.3 0.3 0.4 0.2 0.3 0.3 0.5 0.4 0.7 0.4 0.4 0.7 0.4 0.3 0.5 0.4 0.3 0.6 0.6 0.6 0.7 0.7 0.6 0.7
Ferdousi et al., 2020 [265]	Total = 490 Control = 72 T1D =149 T2D = 269	NDS VPT CDT HDT Sural SNCV	>2	21.6 mm/mm^2^ 23.95 no/mm^2^ 58.54 no/mm^2^	CNFL CNFD CNBD	0.68 0.71 0.70	64 58 69	67 83 65	- - -	- - -	- - -	- - -
Ferdousi et al., 2021 [298]	Control = 72 T1D = 149 T2D = 269	NSP NDS Pinprick VPT CPT HPT DB-HRV NCS Sural SNAP Sural SNCV PMNCV	>2 <40.0 m/s	24 mm/mm^2^ 29.4 no/mm^2^ 64.58 no/mm^2^	CNFL CNFD CNBD	0.73 0.81 0.74	66.7 73.5 66.7	66.4 74.4 66.7	- - -	- - -	- - -	- - -

AUC—area under the curve; CARTs—cardiac autonomic reflex tests; CNBD—corneal nerve fibre branch density; CNFD—corneal nerve fibre density; CNFL—corneal nerve fibre length; CNFT—corneal nerve fibre tortuosity; CPT—cold pain threshold; DAN—diabetic autonomic neuropathy; DB-HRV—deep breathing heart rate variability; DPN—diabetic peripheral neuropathy; HPT—heat pain threshold; IENFD—intra-epithelial nerve fibre density; LDIFlare—laser doppler imager flare; LR+—positive likelihood ratio; LR−—negative likelihood ratio; MNSI—Michigan neuropathy screening instrument; NCS—nerve conduction studies; NDS—Neuropathy Disability Score; NISLL—neuropathy impairment score of the lower limbs; NPV—negative predictive value; NTSS—neuropathy total symptom score; NSP—neuropathy symptom profile; PMNAP—peroneal motor nerve amplitude; PMNCV—peroneal motor nerve conduction velocity; PPV—positive predictive value; QAFT—quantitative autonomic function testing; QSART—quantitative sudomotor axon reflex test; SNAP—sensory nerve action potential; SNCV—sural nerve conduction velocity; T1D—type 1 diabetes; T2D—type 2 diabetes; TCNS—Toronto Clinical Neuropathy Score; TDT—thermal detection thresholds; UENS—Utah Early Neuropathy Scale; VPT—vibration perception threshold. * If used to rule in DPN and is not subsequently compared in a receiver operating characteristic diagnostic efficiency analysis; ** This study used the Toronto grading criteria to rule in DPN using the NDS (none = 0–2, mild = 3–5, moderate 6–8, severe 9–10), NSP, VPT, PMNAP, PMNCV, sural SNAP, sural SNCV and small fibre measures in the absence of abnormal NCS TDT (CDT and HDT; ±2 SD disease threshold compared to control reference values).

## References

[1] Cho N.H., Shaw J.E., Karuranga S., Huang Y., da Rocha Fernandes J.D., Ohlrogge A.W., Malanda B. (2018). IDF Diabetes Atlas: Global estimates of diabetes prevalence for 2017 and projections for 2045. Diabetes Res. Clin. Pract..

[2] Bhutani J., Bhutani S. (2014). Worldwide burden of diabetes. Indian J. Endocrinol. Metab..

[3] Fitzmaurice C., Allen C., Barber R.M., Barregard L., Bhutta Z.A., Brenner H., Dicker D.J., Chimed-Orchir O., Dandona R., Global Burden of Disease Cancer Collaboration (2017). Global, Regional, and National Cancer Incidence, Mortality, Years of Life Lost, Years Lived with Disability, and Disability-Adjusted Life-years for 32 Cancer Groups, 1990 to 2015: A Systematic Analysis for the Global Burden of Disease Study. JAMA Oncol..

[4] Singh R., Kishore L., Kaur N. (2014). Diabetic peripheral neuropathy: Current perspective and future directions. Pharmacol. Res..

[5] Callaghan B.C., Cheng H.T., Stables C.L., Smith A.L., Feldman E.L. (2012). Diabetic neuropathy: Clinical manifestations and current treatments. Lancet Neurol..

[6] Tesfaye S., Boulton A.J.M., Dyck P.J., Freeman R., Horowitz M., Kempler P., Lauria G., Malik R.A., Spallone V., Vinik A. (2010). Diabetic neuropathies: Update on definitions, diagnostic criteria, estimation of severity, and treatments. Diabetes Care.

[7] Pirart J. (1977). Diabetes mellitus and its degenerative complications: A prospective study of 4400 patients observed between 1947 and 1973 (3rd and last part) (author’s transl.). Diabetes Metab..

[8] Gordois A., Scuffham P., Shearer A., Oglesby A., Tobian J.A. (2003). The health care costs of diabetic peripheral neuropathy in the US. Diabetes Care.

[9] Feldman E.L., Callaghan B.C., Pop-Busui R., Zochodne D.W., Wright D.E., Bennett D.L., Bril V., Russell J.W., Viswanathan V. (2019). Diabetic neuropathy. Nat. Rev. Dis. Primers.

[10] Malik R., Veves A., Tesfaye S., Smith G., Cameron N., Zochodne D., Lauria G. (2011). Small Fiber Neuropathy: Role in the diagnosis of Diabetic Sensorimotor Polyneuropathy. Diabetes Metab. Res. Rev..

[11] Breiner A., Lovblom L.E., Perkins B.A., Bril V. (2014). Does the Prevailing Hypothesis That Small-Fiber Dysfunction Precedes Large-Fiber Dysfunction Apply to Type 1 Diabetic Patients?. Diabetes Care.

[12] Ziegler D., Papanas N., Zhivov A., Allgeier S., Winter K., Ziegler I., Bruggemann J., Strom A., Peschel S., Kohler B. (2014). Early detection of nerve fiber loss by corneal confocal microscopy and skin biopsy in recently diagnosed type 2 diabetes. Diabetes.

[13] Dobretsov M., Romanovsky D., Stimers J.R. (2007). Early diabetic neuropathy: Triggers and mechanisms. World J. Gastroenterol..

[14] Azmi S., Jeziorska M., Ferdousi M., Petropoulos I.N., Ponirakis G., Marshall A., Alam U., Asghar O., Atkinson A., Jones W. (2019). Early nerve fibre regeneration in individuals with type 1 diabetes after simultaneous pancreas and kidney transplantation. Diabetologia.

[15] Smith A.G., Russell J., Feldman E.L., Goldstein J., Peltier A., Smith S., Hamwi J., Pollari D., Bixby B., Howard J. (2006). Lifestyle intervention for pre-diabetic neuropathy. Diabetes Care.

[16] Tavakoli M., Mitu-Pretorian M., Petropoulos I.N., Fadavi H., Asghar O., Alam U., Ponirakis G., Jeziorska M., Marshall A., Efron N. (2013). Corneal confocal microscopy detects early nerve regeneration in diabetic neuropathy after simultaneous pancreas and kidney transplantation. Diabetes.

[17] Abbott C.A., Malik R.A., van Ross E.R., Kulkarni J., Boulton A.J. (2011). Prevalence and characteristics of painful diabetic neuropathy in a large community-based diabetic population in the U.K. Diabetes Care.

[18] Veves A., Backonja M., Malik R.A. (2008). Painful diabetic neuropathy: Epidemiology, natural history, early diagnosis, and treatment options. Pain Med..

[19] Pruitt J., Moracho-Vilrriales C., Threatt T., Wagner S., Wu J., Romero-Sandoval E.A. (2017). Identification, prevalence, and treatment of painful diabetic neuropathy in patients from a rural area in South Carolina. J. Pain Res..

[20] Attal N., Lanteri-Minet M., Laurent B., Fermanian J., Bouhassira D. (2011). The specific disease burden of neuropathic pain: Results of a French nationwide survey. Pain.

[21] Finnerup N.B., Attal N., Haroutounian S., McNicol E., Baron R., Dworkin R.H., Gilron I., Haanpää M., Hansson P., Jensen T.S. (2015). Pharmacotherapy for neuropathic pain in adults: A systematic review and meta-analysis. Lancet Neurol..

[22] Quattrini C., Tesfaye S. (2003). Understanding the impact of painful diabetic neuropathy. Diabetes Metab. Res. Rev..

[23] Gore M., Brandenburg N.A., Dukes E., Hoffman D.L., Tai K.-S., Stacey B. (2005). Pain severity in diabetic peripheral neuropathy is associated with patient functioning, symptom levels of anxiety and depression, and sleep. J. Pain Symptom Manag..

[24] Stewart W.F., Ricci J.A., Chee E., Hirsch A.G., Brandenburg N.A. (2007). Lost productive time and costs due to diabetes and diabetic neuropathic pain in the US workforce. J. Occup. Environ. Med..

[25] Scholz J., Finnerup N.B., Attal N., Aziz Q., Baron R., Bennett M.I., Benoliel R., Cohen M., Cruccu G., Davis K.D. (2019). The IASP classification of chronic pain for ICD-11: Chronic neuropathic pain. Pain.

[26] Torrance N., Elliott A.M., Lee A.J., Smith B.H. (2010). Severe chronic pain is associated with increased 10 year mortality. A cohort record linkage study. Eur. J. Pain.

[27] Ziegler D., Strom A., Lobmann R., Reiners K., Rett K., Schnell O. (2015). High prevalence of diagnosed and undiagnosed polyneuropathy in subjects with and without diabetes participating in a nationwide educational initiative (PROTECT study). J. Diabetes Complicat..

[28] Ponirakis G., Elhadd T., Chinnaiyan S., Dabbous Z., Siddiqui M., Al-Muhannadi H., Petropoulos I., Khan A., Ashawesh K.A.E., Dukhan K.M.O. (2019). Prevalence and risk factors for painful diabetic neuropathy in secondary healthcare in Qatar. J. Diabetes Investig..

[29] Ponirakis G., Elhadd T., Chinnaiyan S., Dabbous Z., Siddiqui M., Al-Muhannadi H., Petropoulos I.N., Khan A., Ashawesh K.A.E., Dukhan K.M.O. (2020). Prevalence and management of diabetic neuropathy in secondary care in Qatar. Diabetes Metab. Res. Rev..

[30] Vinik A.I. (2003). Management of neuropathy and foot problems in diabetic patients. Clin. Cornerstone.

[31] Vinik A.I., Park T.S., Stansberry K.B., Pittenger G.L. (2000). Diabetic neuropathies. Diabetologia.

[32] Margolis D.J., Jeffcoate W. (2013). Epidemiology of foot ulceration and amputation: Can global variation be explained?. Med. Clin..

[33] Larsson J., Agardh C.-D., Apelqvist J., Stenström A. (1998). Long-term prognosis after healed amputation in patients with diabetes. Clin. Orthop. Relat. Res..

[34] Tentolouris N., Al-Sabbagh S., Walker M.G., Boulton A.J., Jude E.B. (2004). Mortality in diabetic and nondiabetic patients after amputations performed from 1990 to 1995: A 5-year follow-up study. Diabetes Care.

[35] Icks A., Scheer M., Morbach S., Genz J., Haastert B., Giani G., Glaeske G., Hoffmann F. (2011). Time-dependent impact of diabetes on mortality in patients after major lower extremity amputation: Survival in a population-based 5-year cohort in Germany. Diabetes Care.

[36] Bakker K., Apelqvist J., Lipsky B.A., Van Netten J.J. (2016). The 2015 IWGDF guidance documents on prevention and management of foot problems in diabetes: Development of an evidence-based global consensus. Diabetes Metab. Res. Rev..

[37] Tchero H., Kangambega P., Lin L., Mukisi-Mukaza M., Brunet-Houdard S., Briatte C., Retali G.R., Rusch E. (2018). Cost of diabetic foot in France, Spain, Italy, Germany and United Kingdom: A systematic review. Ann. Endocrinol..

[38] NHS (2014). Annual Diabetes Foot Care Report. https://www.england.nhs.uk/south/wp-content/uploads/sites/6/2017/07/se-clinical-network-foot-care-annual-report-october-2016.pdf.

[39] Feldman E.L., Nave K.A., Jensen T.S., Bennett D.L.H. (2017). New Horizons in Diabetic Neuropathy: Mechanisms, Bioenergetics, and Pain. Neuron.

[40] Cameron N.E., Eaton S.E.M., Cotter M.A., Tesfaye S. (2001). Vascular factors and metabolic interactions in the pathogenesis of diabetic neuropathy. Diabetologia.

[41] Greene D.A., Stevens M.J., Obrosova I., Feldman E.L. (1999). Glucose-induced oxidative stress and programmed cell death in diabetic neuropathy. Eur. J. Pharmacol..

[42] Zhou J., Zhou S. (2014). Inflammation: Therapeutic Targets for Diabetic Neuropathy. Mol. Neurobiol..

[43] Obrosova I.G., Li F., Abatan O.I., Forsell M.A., Komjáti K., Pacher P., Szabó C., Stevens M.J. (2004). Role of poly(ADP-ribose) polymerase activation in diabetic neuropathy. Diabetes.

[44] Obrosova I.G., Drel V.R., Oltman C.L., Mashtalir N., Tibrewala J., Groves J.T., Yorek M.A. (2007). Role of nitrosative stress in early neuropathy and vascular dysfunction in streptozotocin-diabetic rats. Am. J. Physiol. Endocrinol. Metab..

[45] Vareniuk I., Pacher P., Pavlov I.A., Drel V.R., Obrosova I.G. (2009). Peripheral neuropathy in mice with neuronal nitric oxide synthase gene deficiency. Int. J. Mol. Med..

[46] Kobayashi M., Zochodne D.W. (2018). Diabetic neuropathy and the sensory neuron: New aspects of pathogenesis and their treatment implications. J. Diabetes Investig..

[47] Hosseini A., Abdollahi M. (2013). Diabetic neuropathy and oxidative stress: Therapeutic perspectives. Oxidative Med. Cell. Longev..

[48] Lukic I.K., Humpert P.M., Nawroth P.P., Bierhaus A. (2008). The RAGE pathway: Activation and perpetuation in the pathogenesis of diabetic neuropathy. Ann. N. Y. Acad. Sci..

[49] Casellini C.M., Barlow P.M., Rice A.L., Casey M., Simmons K., Pittenger G., Bastyr E.J., Wolka A.M., Vinik A.I. (2007). A 6-month, randomized, double-masked, placebo-controlled study evaluating the effects of the protein kinase C-beta inhibitor ruboxistaurin on skin microvascular blood flow and other measures of diabetic peripheral neuropathy. Diabetes Care.

[50] Jack M., Wright D. (2012). Role of advanced glycation endproducts and glyoxalase I in diabetic peripheral sensory neuropathy. Transl. Res..

[51] Geraldes P., King G.L. (2010). Activation of protein kinase C isoforms and its impact on diabetic complications. Circ. Res..

[52] So K. (2020). Roles of TRPA1 in Painful Dysesthesia. Yakugaku Zasshi.

[53] Pek S.L.T., Lim S.C., Ang K., Kwan P.Y., Tang W.E., Sum C.F., Tavintharan S. (2020). Endothelin-1 predicts incident diabetic peripheral neuropathy in Type 2 Diabetes: A cohort study. Eur. J. Endocrinol..

[54] Tesfaye S., Chaturvedi N., Eaton S.E.M., Ward J.D., Manes C., Ionescu-Tirgoviste C., Witte D.R., Fuller J.H. (2005). Vascular Risk Factors and Diabetic Neuropathy. N. Engl. J. Med..

[55] The DCCT Research Group (1993). The Effect of Intensive Treatment of Diabetes on the Development and Progression of Long-Term Complications in Insulin-Dependent Diabetes Mellitus. N. Engl. J. Med..

[56] The DCCT Research Group (1995). Effect of intensive diabetes treatment on nerve conduction in the Diabetes Control and Complications Trial. Ann. Neurol..

[57] Epidemiology of Diabetes Interventions and Complications (EDIC) (1999). Epidemiology of Diabetes Interventions and Complications (EDIC). Design, implementation, and preliminary results of a long-term follow-up of the Diabetes Control and Complications Trial cohort. Diabetes Care.

[58] Callaghan B.C., Little A.A., Feldman E.L., Hughes R.A.C. (2012). Enhanced glucose control for preventing and treating diabetic neuropathy. Cochrane Database Syst. Rev..

[59] Smith A.G., Singleton J.R. (2013). Obesity and hyperlipidemia are risk factors for early diabetic neuropathy. J. Diabetes Complicat..

[60] Malik R.A., Williamson S., Abbott C., Carrington A.L., Iqbal J., Schady W., Boulton A.J. (1998). Effect of angiotensin-converting-enzyme (ACE) inhibitor trandolapril on human diabetic neuropathy: Randomised double-blind controlled trial. Lancet.

[61] Ponirakis G., Petropoulos I.N., Alam U., Ferdousi M., Asghar O., Marshall A., Azmi S., Jeziorska M., Mahfoud Z.R., Boulton A.J.M. (2019). Hypertension Contributes to Neuropathy in Patients with Type 1 Diabetes. Am. J. Hypertens..

[62] Ruggenenti P., Lauria G., Iliev I.P., Fassi A., Ilieva A.P., Rota S., Chiurchiu C., Barlovic D.P., Sghirlanzoni A., Lombardi R. (2011). Effects of manidipine and delapril in hypertensive patients with type 2 diabetes mellitus: The delapril and manidipine for nephroprotection in diabetes (DEMAND) randomized clinical trial. Hypertension.

[63] Hernández-Ojeda J., Román-Pintos L.M., Rodríguez-Carrízalez A.D., Troyo-Sanromán R., Cardona-Muñoz E.G., Alatorre-Carranza M.D.P., Miranda-Díaz A.G. (2014). Effect of rosuvastatin on diabetic polyneuropathy: A randomized, double-blind, placebo-controlled Phase IIa study. Diabetes Metab. Syndr. Obes. Targets Ther..

[64] Balducci S., Iacobellis G., Parisi L., Di Biase N., Calandriello E., Leonetti F., Fallucca F. (2006). Exercise training can modify the natural history of diabetic peripheral neuropathy. J. Diabetes Complicat..

[65] Kluding P.M., Pasnoor M., Singh R., Jernigan S., Farmer K., Rucker J., Sharma N.K., Wright D.E. (2012). The effect of exercise on neuropathic symptoms, nerve function, and cutaneous innervation in people with diabetic peripheral neuropathy. J. Diabetes Complicat..

[66] Malik R. (2008). Neuropad: Early diagnostic test for diabetic peripheral neuropathy. Prescriber.

[67] NICE, National Institute for Health and Care Eexelence Diabetic Foot Problems: Prevention and Management, NICE Guideline (NG19). https://www.nice.org.uk/guidance/ng19.

[68] Kamei N., Yamane K., Nakanishi S., Yamashita Y., Tamura T., Ohshita K., Watanabe H., Fujikawa R., Okubo M., Kohno N. (2005). Effectiveness of Semmes–Weinstein monofilament examination for diabetic peripheral neuropathy screening. J. Diabetes Complicat..

[69] Young M.J., Breddy J.L., Veves A., Boulton A.J.M. (1994). The Prediction of Diabetic Neuropathic Foot Ulceration Using Vibration Perception Thresholds: A prospective study. Diabetes Care.

[70] Tan L.S. (2010). The clinical use of the 10 g monofilament and its limitations: A review. Diabetes Res. Clin. Pract..

[71] Rith-Najarian S.J., Stolusky T., Gohdes D.M. (1992). Identifying diabetic patients at high risk for lower-extremity amputation in a primary health care setting. A prospective evaluation of simple screening criteria. Diabetes Care.

[72] Bansal V., Kalita J., Misra U.K. (2006). Diabetic neuropathy. Postgrad. Med. J..

[73] Liew G., Michaelides M., Bunce C. (2014). A comparison of the causes of blindness certifications in England and Wales in working age adults (16–64 years), 1999–2000 with 2009–2010. BMJ Open.

[74] Marshall S.M. (2012). Diabetic nephropathy in type 1 diabetes: Has the outlook improved since the 1980s?. Diabetologia.

[75] Flaxman S.R., Bourne R.R.A., Resnikoff S., Ackland P., Braithwaite T., Cicinelli M.V., Das A., Jonas J.B., Keeffe J., Kempen J.H. (2017). Global causes of blindness and distance vision impairment 1990–2020: A systematic review and meta-analysis. Lancet Glob. Health.

[76] Landowski L.M., Dyck P.J.B., Engelstad J., Taylor B.V. (2016). Axonopathy in peripheral neuropathies: Mechanisms and therapeutic approaches for regeneration. J. Chem. Neuroanat..

[77] Gæde P., Oellgaard J., Carstensen B., Rossing P., Lund-Andersen H., Parving H.-H., Pedersen O. (2016). Years of life gained by multifactorial intervention in patients with type 2 diabetes mellitus and microalbuminuria: 21 years follow-up on the Steno-2 randomised trial. Diabetologia.

[78] Pop-Busui R., Boulton A.J.M., Feldman E.L., Bril V., Freeman R., Malik R.A., Sosenko J.M., Ziegler D. (2017). Diabetic Neuropathy: A Position Statement by the American Diabetes Association. Diabetes Care.

[79] Perkins B.A., Olaleye D., Zinman B., Bril V. (2001). Simple Screening Tests for Peripheral Neuropathy in the Diabetes Clinic. Diabetes Care.

[80] Britland S.T., Young R.J., Sharma A.K., Clarke B.F. (1990). Association of painful and painless diabetic polyneuropathy with different patterns of nerve fiber degeneration and regeneration. Diabetes.

[81] Dyck P.J., Sherman W.R., Hallcher L.M., Service F.J., O’Brien P.C., Grina L.A., Palumbo P.J., Swanson C.J. (1980). Human diabetic endoneurial sorbitol, fructose, and myo-inositol related to sural nerve morphometry. Ann. Neurol..

[82] Dyck P.J., Davies J.L., Litchy W.J., O’Brien P.C. (1997). Longitudinal assessment of diabetic polyneuropathy using a composite score in the Rochester Diabetic Neuropathy Study cohort. Neurology.

[83] England J.D., Gronseth G.S., Franklin G., Miller R.G., Asbury A.K., Carter G.T., Cohen J.A., Fisher M.A., Howard J.F., Kinsella L.J. (2005). Distal symmetric polyneuropathy: A definition for clinical research. A Report of the American Academy of Neurology, the American Association of Electrodiagnostic Medicine, and the American Academy of Physical Medicine and Rehabilitation. Neurology.

[84] Feldman E.L., Stevens M.J., Thomas P.K., Brown M.B., Canal N., Greene D.A. (1994). A practical two-step quantitative clinical and electrophysiological assessment for the diagnosis and staging of diabetic neuropathy. Diabetes Care.

[85] Herman W.H., Pop-Busui R., Braffett B.H., Martin C.L., Cleary P.A., Albers J.W., Feldman E.L., Group D.E.R. (2012). Use of the Michigan Neuropathy Screening Instrument as a measure of distal symmetrical peripheral neuropathy in Type 1 diabetes: Results from the Diabetes Control and Complications Trial/Epidemiology of Diabetes Interventions and Complications. Diabet. Med. J. Br. Diabet. Assoc..

[86] Andersen S.T., Witte D.R., Dalsgaard E.M., Andersen H., Nawroth P., Fleming T., Jensen T.M., Finnerup N.B., Jensen T.S., Lauritzen T. (2018). Risk Factors for Incident Diabetic Polyneuropathy in a Cohort with Screen-Detected Type 2 Diabetes Followed for 13 Years: ADDITION-Denmark. Diabetes Care.

[87] Mete T., Aydin Y., Saka M., Cinar Yavuz H., Bilen S., Yalcin Y., Arli B., Berker D., Guler S. (2013). Comparison of Efficiencies of Michigan Neuropathy Screening Instrument, Neurothesiometer, and Electromyography for Diagnosis of Diabetic Neuropathy. Int. J. Endocrinol..

[88] Dyck P.J., Litchy W.J., Lehman K.A., Hokanson J.L., Low P.A., O’Brien P.C. (1995). Variables influencing neuropathic endpoints: The Rochester Diabetic Neuropathy Study of Healthy Subjects. Neurology.

[89] Weintrob N., Amitay I., Lilos P., Shalitin S., Lazar L., Josefsberg Z. (2007). Bedside neuropathy disability score compared to quantitative sensory testing for measurement of diabetic neuropathy in children, adolescents, and young adults with type 1 diabetes. J. Diabetes Complicat..

[90] Meijer J.W., Bosma E., Lefrandt J.D., Links T.P., Smit A.J., Stewart R.E., Van Der Hoeven J.H., Hoogenberg K. (2003). Clinical diagnosis of diabetic polyneuropathy with the diabetic neuropathy symptom and diabetic neuropathy examination scores. Diabetes Care.

[91] Bril V. (1999). NIS-LL: The primary measurement scale for clinical trial endpoints in diabetic peripheral neuropathy. Eur. Neurol..

[92] Davies M., Brophy S., Williams R., Taylor A. (2006). The prevalence, severity, and impact of painful diabetic peripheral neuropathy in type 2 diabetes. Diabetes Care.

[93] Krumova E.K., Geber C., Westermann A., Maier C. (2012). Neuropathic pain: Is quantitative sensory testing helpful?. Curr. Diabetes Rep..

[94] Shy M.E., Frohman E.M., So Y.T., Arezzo J.C., Cornblath D.R., Giuliani M.J., Kincaid J.C., Ochoa J.L., Parry G.J., Weimer L.H. (2003). Quantitative sensory testing: Report of the Therapeutics and Technology Assessment Subcommittee of the AAN. Neurology.

[95] Gruener G., Dyck P.J. (1994). Quantitative sensory testing: Methodology, applications, and future directions. J. Clin. Neurophysiol..

[96] Vlckova-Moravcova E., Bednarik J., Belobradkova J., Sommer C. (2008). Small-fibre involvement in diabetic patients with neuropathic foot pain. Diabet. Med..

[97] Jimenez-Cohl P., Grekin C., Leyton C., Vargas C., Villaseca R. (2012). Thermal Threshold: Research Study on Small Fiber Dysfunction in Distal Diabetic Polyneuropathy. J. Diabetes Sci. Technol..

[98] Chao C.C., Hsieh S.C., Yang W.S., Lin Y.H., Lin W.M., Tai T.Y., Hsieh S.T. (2007). Glycemic control is related to the severity of impaired thermal sensations in type 2 diabetes. Diabetes Metab. Res. Rev..

[99] Bonhof G.J., Strom A., Puttgen S., Ringel B., Bruggemann J., Bodis K., Mussig K., Szendroedi J., Roden M., Ziegler D. (2017). Patterns of cutaneous nerve fibre loss and regeneration in type 2 diabetes with painful and painless polyneuropathy. Diabetologia.

[100] Krämer H.H., Rolke R., Bickel A., Birklein F. (2004). Thermal thresholds predict painfulness of diabetic neuropathies. Diabetes Care.

[101] Alam U., Jeziorska M., Petropoulos I.N., Asghar O., Fadavi H., Ponirakis G., Marshall A., Tavakoli M., Boulton A.J.M., Efron N. (2017). Diagnostic utility of corneal confocal microscopy and intra-epidermal nerve fibre density in diabetic neuropathy. PLoS ONE.

[102] Nebuchennykh M., Løseth S., Lindal S., Mellgren S.I. (2009). The value of skin biopsy with recording of intraepidermal nerve fiber density and quantitative sensory testing in the assessment of small fiber involvement in patients with different causes of polyneuropathy. J. Neurol..

[103] Abraham A., Alabdali M., Alsulaiman A., Albulaihe H., Breiner A., Katzberg H.D., Aljaafari D., Lovblom L.E., Bril V. (2017). The sensitivity and specificity of the neurological examination in polyneuropathy patients with clinical and electrophysiological correlations. PLoS ONE.

[104] Vinik A., Casselini C., Nevoret M.-L., Fiengold K.R., Anawalt B., Boyce A., Chrousos G., de Herder W.W., Dungan K., Grossman A. Diabetic Neuropathies. https://www.ncbi.nlm.nih.gov/books/NBK279175/.

[105] Santos T.R.M., Melo J.V., Leite N.C., Salles G.F., Cardoso C.R.L. (2018). Usefulness of the vibration perception thresholds measurement as a diagnostic method for diabetic peripheral neuropathy: Results from the Rio de Janeiro type 2 diabetes cohort study. J. Diabetes Complicat..

[106] Martin C.L., Waberski B.H., Pop-Busui R., Cleary P.A., Catton S., Albers J.W., Feldman E.L., Herman W.H. (2010). Vibration perception threshold as a measure of distal symmetrical peripheral neuropathy in type 1 diabetes: Results from the DCCT/EDIC study. Diabetes Care.

[107] Bril V., Perkins B.A. (2002). Comparison of vibration perception thresholds obtained with the Neurothesiometer and the CASE IV and relationship to nerve conduction studies. Diabet. Med..

[108] Levy D.M., Abraham R.R., Abraham R.M. (1987). Small- and large-fiber involvement in early diabetic neuropathy: A study with the medial plantar response and sensory thresholds. Diabetes Care.

[109] Armstrong F.M., Bradbury J.E., Ellis S.H., Owens D.R., Rosen I., Sonksen P., Sundkvist G. (1991). A study of peripheral diabetic neuropathy. The application of age-related reference values. Diabet. Med..

[110] Maier C., Baron R., Tölle T.R., Binder A., Birbaumer N., Birklein F., Gierthmühlen J., Flor H., Geber C., Huge V. (2010). Quantitative sensory testing in the German Research Network on Neuropathic Pain (DFNS): Somatosensory abnormalities in 1236 patients with different neuropathic pain syndromes. Pain.

[111] Moloney N.A., Hall T.M., Doody C.M. (2012). Reliability of thermal quantitative sensory testing: A systematic review. J. Rehabil. Res. Dev..

[112] Chong P.S., Cros D.P. (2004). Technology literature review: Quantitative sensory testing. Muscle Nerve.

[113] Morrison I., Loken L.S., Minde J., Wessberg J., Perini I., Nennesmo I., Olausson H. (2011). Reduced C-afferent fibre density affects perceived pleasantness and empathy for touch. Brain J. Neurol..

[114] Rolke R., Baron R., Maier C., Tölle T.R., Treede R.D., Beyer A., Binder A., Birbaumer N., Birklein F., Bötefür I.C. (2006). Quantitative sensory testing in the German Research Network on Neuropathic Pain (DFNS): Standardized protocol and reference values. Pain.

[115] Marcuzzi A., Wrigley P.J., Dean C.M., Adams R., Hush J.M. (2017). The long-term reliability of static and dynamic quantitative sensory testing in healthy individuals. Pain.

[116] Magerl W., Ali Z., Ellrich J., Meyer R.A., Treede R.D. (1999). C- and A delta-fiber components of heat-evoked cerebral potentials in healthy human subjects. Pain.

[117] Fruhstorfer H. (1984). Thermal sensibility changes during ischemic nerve block. Pain.

[118] Magerl W., Krumova E.K., Baron R., Tolle T., Treede R.-D., Maier C. (2010). Reference data for quantitative sensory testing (QST): Refined stratification for age and a novel method for statistical comparison of group data. Pain.

[119] Blankenburg M., Boekens H., Hechler T., Maier C., Krumova E., Scherens A., Magerl W., Aksu F., Zernikow B. (2010). Reference values for quantitative sensory testing in children and adolescents: Developmental and gender differences of somatosensory perception. Pain.

[120] Pfau D.B., Krumova E.K., Treede R.D., Baron R., Toelle T., Birklein F., Eich W., Geber C., Gerhardt A., Weiss T. (2014). Quantitative sensory testing in the German Research Network on Neuropathic Pain (DFNS): Reference data for the trunk and application in patients with chronic postherpetic neuralgia. Pain.

[121] Backonja M.M., Attal N., Baron R., Bouhassira D., Drangholt M., Dyck P.J., Edwards R.R., Freeman R., Gracely R., Haanpaa M.H. (2013). Value of quantitative sensory testing in neurological and pain disorders: NeuPSIG consensus. Pain.

[122] Hansson P., Backonja M., Bouhassira D. (2007). Usefulness and limitations of quantitative sensory testing: Clinical and research application in neuropathic pain states. Pain.

[123] Yarnitsky D., Bouhassira D., Drewes A.M., Fillingim R.B., Granot M., Hansson P., Landau R., Marchand S., Matre D., Nilsen K.B. (2015). Recommendations on practice of conditioned pain modulation (CPM) testing. Eur. J. Pain.

[124] Kopf S., Groener J.B., Kender Z., Fleming T., Bischoff S., Jende J., Schumann C., Ries S., Bendszus M., Schuh-Hofer S. (2018). Deep phenotyping neuropathy: An underestimated complication in patients with pre-diabetes and type 2 diabetes associated with albuminuria. Diabetes Res. Clin. Pract..

[125] Üçeyler N., Vollert J., Broll B., Riediger N., Langjahr M., Saffer N., Schubert A.L., Siedler G., Sommer C. (2018). Sensory profiles and skin innervation of patients with painful and painless neuropathies. Pain.

[126] Scherens A., Maier C., Haussleiter I.S., Schwenkreis P., Vlckova-Moravcova E., Baron R., Sommer C. (2009). Painful or painless lower limb dysesthesias are highly predictive of peripheral neuropathy: Comparison of different diagnostic modalities. Eur. J. Pain.

[127] Schmelz M. (2020). What can we learn from the failure of QST?. Pain.

[128] Blesneac I., Themistocleous A.C., Fratter C., Conrad L.J., Ramirez J.D., Cox J.J., Tesfaye S., Shillo P.R., Rice A.S.C., Tucker S.J. (2018). Rare NaV1.7 variants associated with painful diabetic peripheral neuropathy. Pain.

[129] Haanpää M., Attal N., Backonja M., Baron R., Bennett M., Bouhassira D., Cruccu G., Hansson P., Haythornthwaite J.A., Iannetti G.D. (2011). NeuPSIG guidelines on neuropathic pain assessment. Pain.

[130] Backonja M.M., Walk D., Edwards R.R., Sehgal N., Moeller-Bertram T., Wasan A., Irving G., Argoff C., Wallace M. (2009). Quantitative sensory testing in measurement of neuropathic pain phenomena and other sensory abnormalities. Clin. J. Pain.

[131] Cruccu G., Truini A. (2009). Tools for assessing neuropathic pain. PLoS Med..

[132] Pfau D.B., Geber C., Birklein F., Treede R.-D. (2012). Quantitative sensory testing of neuropathic pain patients: Potential mechanistic and therapeutic implications. Curr. Pain Headache Rep..

[133] Devigili G., Tugnoli V., Penza P., Camozzi F., Lombardi R., Melli G., Broglio L., Granieri E., Lauria G. (2008). The diagnostic criteria for small fibre neuropathy: From symptoms to neuropathology. Brain J. Neurol..

[134] Lauria G., Lombardi R., Borgna M., Penza P., Bianchi R., Savino C., Canta A., Nicolini G., Marmiroli P., Cavaletti G. (2005). Intraepidermal nerve fiber density in rat foot pad: Neuropathologic-neurophysiologic correlation. J. Peripher. Nerv. Syst..

[135] Vollert J., Attal N., Baron R., Freynhagen R., Haanpää M., Hansson P., Jensen T.S., Rice A.S., Segerdahl M., Serra J. (2016). Quantitative sensory testing using DFNS protocol in Europe: An evaluation of heterogeneity across multiple centers in patients with peripheral neuropathic pain and healthy subjects. Pain.

[136] Malik R.A. (2020). Diabetic neuropathy: A focus on small fibres. Diabetes Metab. Res. Rev..

[137] Cruccu G., Anand P., Attal N., Garcia-Larrea L., Haanpää M., Jørum E., Serra J., Jensen T.S. (2004). EFNS guidelines on neuropathic pain assessment. Eur. J. Neurol..

[138] He G., Wang A., Liu Y., Sheng H., Guo L., Hu D., Yuan S., Hoeijmakers J.G., Faber C.G., Lauria G. (2012). Small-fibre neuropathies—advances in diagnosis, pathophysiology and management. Nat. Rev. Neurol..

[139] Cruccu G., Aminoff M.J., Curio G., Guerit J.M., Kakigi R., Mauguiere F., Rossini P.M., Treede R.D., Garcia-Larrea L. (2008). Recommendations for the clinical use of somatosensory-evoked potentials. Clin. Neurophysiol..

[140] Di Stefano G., La Cesa S., Leone C., Pepe A., Galosi E., Fiorelli M., Valeriani M., Lacerenza M., Pergolini M., Biasiotta A. (2017). Diagnostic accuracy of laser-evoked potentials in diabetic neuropathy. Pain.

[141] Wu S.W., Wang Y.C., Hsieh P.C., Tseng M.T., Chiang M.C., Chu C.P., Feng F.P., Lin Y.H., Hsieh S.T., Chao C.C. (2017). Biomarkers of neuropathic pain in skin nerve degeneration neuropathy: Contact heat-evoked potentials as a physiological signature. Pain.

[142] Granovsky Y., Anand P., Nakae A., Nascimento O., Smith B., Sprecher E., Valls-Solé J. (2016). Normative data for Aδ contact heat evoked potentials in adult population: A multicenter study. Pain.

[143] Lagerburg V., Bakkers M., Bouwhuis A., Hoeijmakers J.G., Smit A.M., Van Den Berg S.J., Hordijk-De Boer I., Brouwer-Van Der Lee M.D., Kranendonk D., Reulen J.P. (2015). Contact heat evoked potentials: Normal values and use in small-fiber neuropathy. Muscle Nerve.

[144] Jutzeler C.R., Rosner J., Rinert J., Kramer J.L.K., Curt A. (2016). Normative data for the segmental acquisition of contact heat evoked potentials in cervical dermatomes. Sci. Rep..

[145] Chao C.C., Tseng M.T., Lin Y.J., Yang W.S., Hsieh S.C., Lin Y.H., Chiu M.J., Chang Y.C., Hsieh S.T. (2010). Pathophysiology of neuropathic pain in type 2 diabetes: Skin denervation and contact heat-evoked potentials. Diabetes Care.

[146] Zhang C., Xie B., Li X., Yao Y. (2014). Contact heat-evoked potentials as a useful means in patients with Guillain-Barré syndrome. Neurol. Sci..

[147] Schestatsky P., Lladó-Carbó E., Casanova-Molla J., Alvarez-Blanco S., Valls-Solé J. (2008). Small fibre function in patients with meralgia paresthetica. Pain.

[148] Parson H.K., Nguyen V.T., Orciga M.A., Boyd A.L., Casellini C.M., Vinik A.I. (2013). Contact heat-evoked potential stimulation for the evaluation of small nerve fiber function. Diabetes Technol. Ther..

[149] Atherton D.D., Facer P., Roberts K.M., Misra V.P., Chizh B.A., Bountra C., Anand P. (2007). Use of the novel Contact Heat Evoked Potential Stimulator (CHEPS) for the assessment of small fibre neuropathy: Correlations with skin flare responses and intra-epidermal nerve fibre counts. BMC Neurol..

[150] Casanova-Molla J., Grau-Junyent J.M., Morales M., Valls-Solé J. (2011). On the relationship between nociceptive evoked potentials and intraepidermal nerve fiber density in painful sensory polyneuropathies. Pain.

[151] Rage M., Van Acker N., Knaapen M.W., Timmers M., Streffer J., Hermans M.P., Sindic C., Meert T., Plaghki L. (2011). Asymptomatic small fiber neuropathy in diabetes mellitus: Investigations with intraepidermal nerve fiber density, quantitative sensory testing and laser-evoked potentials. J. Neurol..

[152] Vallbo Å.B., Hagbarth K.E. (1968). Activity from skin mechanoreceptors recorded percutaneously in awake human subjects. Exp. Neurol..

[153] Bostock H., Campero M., Serra J., Ochoa J. (2003). Velocity recovery cycles of C fibres innervating human skin. J. Physiol..

[154] Bostock H., Campero M., Serra J., Ochoa J.L. (2005). Temperature-dependent double spikes in C-nociceptors of neuropathic pain patients. Brain J. Neurol..

[155] Schmidt R., Kleggetveit I.P., Namer B., Helås T., Obreja O., Schmelz M., Jørum E. (2012). Double spikes to single electrical stimulation correlates to spontaneous activity of nociceptors in painful neuropathy patients. Pain.

[156] Kleggetveit I.P., Namer B., Schmidt R., Helås T., Rückel M., Ørstavik K., Schmelz M., Jørum E. (2012). High spontaneous activity of C-nociceptors in painful polyneuropathy. Pain.

[157] He G., Wang A., Liu Y., Sheng H., Guo L., Hu D., Yuan S., Serra J. (2012). Microneurography: Towards a biomarker of spontaneous pain. Pain.

[158] Serra J., Duan W.R., Locke C., Sola R., Liu W., Nothaft W. (2015). Effects of a T-type calcium channel blocker, ABT-639, on spontaneous activity in C-nociceptors in patients with painful diabetic neuropathy: A randomized controlled trial. Pain.

[159] Campbell J.N., Meyer R.A. (2006). Mechanisms of neuropathic pain. Neuron.

[160] Dubin A.E., Patapoutian A. (2010). Nociceptors: The sensors of the pain pathway. J. Clin. Investig..

[161] Inceu G.V., Veresiu I.A. (2015). Measurement of current perception thresholds using the Neurometer®—applicability in diabetic neuropathy. Clujul Med..

[162] Masson E.A., Veves A., Fernando D., Boulton A.J. (1989). Current perception thresholds: A new, quick, and reproducible method for the assessment of peripheral neuropathy in diabetes mellitus. Diabetologia.

[163] Cheng W.-Y., Jiang Y.D., Chuang L.M., Huang C.-N., Heng L.-T., Wu H.-P., Tai T.-Y., Lin B.J. (1999). Quantitative sensory testing and risk factors of diabetic sensory neuropathy. J. Neurol..

[164] Matsutomo R., Takebayashi K., Aso Y. (2005). Assessment of Peripheral Neuropathy Using Measurement of the Current Perception Threshold with the Neurometer® in Patients with Type 2 Diabetes Mellitus. J. Int. Med. Res..

[165] Lv S.L., Fang C., Hu J., Huang Y., Yang B., Zou R., Wang F.Y., Zhao H.Q. (2015). Assessment of Peripheral Neuropathy Using Measurement of the Current Perception Threshold with the Neurometer® in patients with type 1 diabetes mellitus. Diabetes Res. Clin. Pract..

[166] Yin H., Liu M., Zhu Y., Cui L. (2018). Reference Values and Influencing Factors Analysis for Current Perception Threshold Testing Based on Study of 166 Healthy Chinese. Front. Neurosci..

[167] NeuroMetrix Inc (2013). NC-stat DPN Check Device User Manual.

[168] Neurometrix Inc FDA and Other Governmental Regulation. https://www.sec.gov/Archives/edgar/data/1289850/000162828019000527/a10knuro20181231.htm.

[169] Pafili K., Maltezos E., Papanas N. (2017). NC-stat for the diagnosis of diabetic polyneuropathy. Expert Rev. Med. Devices.

[170] Shibata Y., Himeno T., Kamiya T., Tani H., Nakayama T., Kojima C., Sugiura-Roth Y., Naito E., Kondo M., Tsunekawa S. (2019). Validity and reliability of a point-of-care nerve conduction device in diabetes patients. J. Diabetes Investig..

[171] Lee J.A., Halpern E.M., Lovblom L.E., Yeung E., Bril V., Perkins B.A. (2014). Reliability and validity of a point-of-care sural nerve conduction device for identification of diabetic neuropathy. PLoS ONE.

[172] Chatzikosma G., Pafili K., Demetriou M., Vadikolias K., Maltezos E., Papanas N. (2016). Evaluation of sural nerve automated nerve conduction study in the diagnosis of peripheral neuropathy in patients with type 2 diabetes mellitus. Arch. Med. Sci..

[173] Perkins B.A., Orszag A., Grewal J., Ng E., Ngo M., Bril V. (2008). Multi-site testing with a point-of-care nerve conduction device can be used in an algorithm to diagnose diabetic sensorimotor polyneuropathy. Diabetes Care.

[174] Perkins B.A., Grewal J., Ng E., Ngo M., Bril V. (2006). Validation of a novel point-of-care nerve conduction device for the detection of diabetic sensorimotor polyneuropathy. Diabetes Care.

[175] Scarr D., Lovblom L.E., Cardinez N., Orszag A., Farooqi M.A., Boulet G., Weisman A., Lovshin J.A., Ngo M., Paul N. (2018). Validity of a point-of-care nerve conduction device for polyneuropathy identification in older adults with diabetes: Results from the Canadian Study of Longevity in Type 1 Diabetes. PLoS ONE.

[176] Sharma S., Vas P.R., Rayman G. (2015). Assessment of diabetic neuropathy using a point-of-care nerve conduction device shows significant associations with the LDIFLARE method and clinical neuropathy scoring. J. Diabetes Sci. Technol..

[177] Lauria G., Hsieh S.T., Johansson O., Kennedy W.R., Leger J.M., Mellgren S.I., Nolano M., Merkies I.S., Polydefkis M., Smith A.G. (2010). European Federation of Neurological Societies/Peripheral Nerve Society Guideline on the use of skin biopsy in the diagnosis of small fiber neuropathy. Report of a joint task force of the European Federation of Neurological Societies and the Peripheral Nerve Society. Eur. J. Neurol..

[178] Lauria G., Lombardi R. (2007). Skin biopsy: A new tool for diagnosing peripheral neuropathy. BMJ.

[179] Chien H.-F., Tseng T.-J., Lin W.-M., Yang C.-C., Chang Y.-C., Chen R.-C., Hsieh S.-T. (2001). Quantitative pathology of cutaneous nerve terminal degeneration in the human skin. Acta Neuropathol..

[180] Collongues N., Samama B., Schmidt-Mutter C., Chamard-Witkowski L., Debouverie M., Chanson J.-B., Antal M.-C., Benardais K., de Seze J., Velten M. (2018). Quantitative and qualitative normative dataset for intraepidermal nerve fibers using skin biopsy. PLoS ONE.

[181] Lauria G., Bakkers M., Schmitz C., Lombardi R., Penza P., Devigili G., Smith A.G., Hsieh S.-T., Mellgren S.I., Umapathi T. (2010). Intraepidermal nerve fiber density at the distal leg: A worldwide normative reference study. J. Peripher. Nerv. Syst..

[182] Løseth S., Stålberg E., Jorde R., Mellgren S.I. (2008). Early diabetic neuropathy: Thermal thresholds and intraepidermal nerve fibre density in patients with normal nerve conduction studies. J. Neurol..

[183] Myers M.I., Peltier A.C. (2013). Uses of skin biopsy for sensory and autonomic nerve assessment. Curr. Neurol. Neurosci. Rep..

[184] Smith S.M., Dworkin R.H., Turk D.C., Baron R., Polydefkis M., Tracey I., Borsook D., Edwards R.R., Harris R.E., Wager T.D. (2017). The Potential Role of Sensory Testing, Skin Biopsy, and Functional Brain Imaging as Biomarkers in Chronic Pain Clinical Trials: IMMPACT Considerations. J. Pain.

[185] Vlčková-Moravcová E., Bednařík J., Dušek L., Toyka K.V., Sommer C. (2008). Diagnostic validity of epidermal nerve fiber densities in painful sensory neuropathies. Muscle Nerve.

[186] Quattrini C., Tavakoli M., Jeziorska M., Kallinikos P., Tesfaye S., Finnigan J., Marshall A., Boulton A.J.M., Efron N., Malik R.A. (2007). Surrogate markers of small fiber damage in human diabetic neuropathy. Diabetes.

[187] Pittenger G.L., Ray M., Burcus N.I., McNulty P., Basta B., Vinik A.I. (2004). Intraepidermal nerve fibers are indicators of small-fiber neuropathy in both diabetic and nondiabetic patients. Diabetes Care.

[188] Shun C.T., Chang Y.C., Wu H.P., Hsieh S.C., Lin W.M., Lin Y.H., Tai T.Y., Hsieh S.T. (2004). Skin denervation in type 2 diabetes: Correlations with diabetic duration and functional impairments. Brain.

[189] Koskinen M., Hietaharju A., Kylaniemi M., Peltola J., Rantala I., Udd B., Haapasalo H. (2005). A quantitative method for the assessment of intraepidermal nerve fibers in small-fiber neuropathy. J. Neurol..

[190] McArthur J.C., Stocks E.A., Hauer P., Cornblath D.R., Griffin J.W. (1998). Epidermal nerve fiber density: Normative reference range and diagnostic efficiency. Arch. Neurol..

[191] Sorensen L., Molyneaux L., Yue D.K. (2006). The relationship among pain, sensory loss, and small nerve fibers in diabetes. Diabetes Care.

[192] Krishnan S.T., Quattrini C., Jeziorska M., Malik R.A., Rayman G. (2009). Abnormal LDIflare but Normal Quantitative Sensory Testing and Dermal Nerve Fiber Density in Patients with Painful Diabetic Neuropathy. Diabetes Care.

[193] Singleton J.R., Marcus R.L., Lessard M.K., Jackson J.E., Smith A.G. (2015). Supervised exercise improves cutaneous reinnervation capacity in metabolic syndrome patients. Ann. Neurol..

[194] Cheng H.T., Dauch J.R., Porzio M.T., Yanik B.M., Hsieh W., Smith A.G., Singleton J.R., Feldman E.L. (2013). Increased axonal regeneration and swellings in intraepidermal nerve fibers characterize painful phenotypes of diabetic neuropathy. J. Pain.

[195] Scheytt S., Riediger N., Braunsdorf S., Sommer C., Üçeyler N. (2015). Increased gene expression of growth associated protein-43 in skin of patients with early-stage peripheral neuropathies. J. Neurol. Sci..

[196] Polydefkis M., Hauer P., Sheth S., Sirdofsky M., Griffin J.W., McArthur J.C. (2004). The time course of epidermal nerve fibre regeneration: Studies in normal controls and in people with diabetes, with and without neuropathy. Brain J. Neurol..

[197] Petropoulos I.N., Ponirakis G., Khan A., Almuhannadi H., Gad H., Malik R.A. (2018). Diagnosing Diabetic Neuropathy: Something Old, Something New. Diabetes Metab. J..

[198] Mayaudon H., Miloche P.O., Bauduceau B. (2010). A new simple method for assessing sudomotor function: Relevance in type 2 diabetes. Diabetes Metab..

[199] Yang Z., Xu B., Lu J., Tian X., Li M., Sun K., Huang F., Liu Y., Xu M., Bi Y. (2013). Autonomic test by EZSCAN in the screening for prediabetes and diabetes. PLoS ONE.

[200] Eh Schwarz P., Brunswick P., Calvet J.-H. (2011). EZSCAN™ a new technology to detect diabetes risk. Br. J. Diabetes Vasc. Dis..

[201] Chen X., Chen L., Ding R., Shi Q., Zhang Y., Hu D. (2015). A preliminary investigation of EZSCAN™ screening for impaired glucose tolerance and diabetes in a patient population. Exp. Ther. Med..

[202] Bernabe-Ortiz A., Ruiz-Alejos A., Miranda J.J., Mathur R., Perel P., Smeeth L. (2017). EZSCAN for undiagnosed type 2 diabetes mellitus: A systematic review and meta-analysis. PLoS ONE.

[203] Zhu L., Zhao X., Zeng P., Zhu J., Yang S., Liu A., Song Y. (2016). Study on autonomic dysfunction and metabolic syndrome in Chinese patients. J. Diabetes Investig..

[204] Sarita B., Alankar T., Ajeet Kumar C., Rameshwar Prasad S. (2016). Detection of Microvascular Complications of Type 2 Diabetes by Ezscan and Its Comparison with Standard Screening Methods. J. Evid. Based Med. Healthc..

[205] Sudoscan SUDOSCAN Approved by the US Food and Drug Administration. https://www.impeto-medical.com/sudoscan-approved-by-the-us-food-and-drug-administration/.

[206] Sumner C.J., Sheth S., Griffin J.W., Cornblath D.R., Polydefkis M. (2003). The spectrum of neuropathy in diabetes and impaired glucose tolerance. Neurology.

[207] Casellini C.M., Parson H.K., Richardson M.S., Nevoret M.L., Vinik A.I. (2013). Sudoscan, a noninvasive tool for detecting diabetic small fiber neuropathy and autonomic dysfunction. Diabetes Technol. Ther..

[208] Selvarajah D., Cash T., Davies J., Sankar A., Rao G., Grieg M., Pallai S., Gandhi R., Wilkinson I.D., Tesfaye S. (2015). SUDOSCAN: A Simple, Rapid, and Objective Method with Potential for Screening for Diabetic Peripheral Neuropathy. PLoS ONE.

[209] Mao F., Liu S., Qiao X., Zheng H., Xiong Q., Wen J., Liu L., Tang M., Zhang S., Zhang Z. (2017). Sudoscan is an effective screening method for asymptomatic diabetic neuropathy in Chinese type 2 diabetes mellitus patients. J. Diabetes Investig..

[210] Goel A., Shivaprasad C., Kolly A., Sarathi H.A.V., Atluri S. (2017). Comparison of electrochemical skin conductance and vibration perception threshold measurement in the detection of early diabetic neuropathy. PLoS ONE.

[211] Smith A.G., Lessard M., Reyna S., Doudova M., Singleton J.R. (2014). The diagnostic utility of Sudoscan for distal symmetric peripheral neuropathy. J. Diabetes Complicat..

[212] Rajan S., Campagnolo M., Callaghan B., Gibbons C.H. (2019). Sudomotor function testing by electrochemical skin conductance: Does it really measure sudomotor function?. Clin. Auton. Res..

[213] Papanas N., Papatheodorou K., Christakidis D., Papazoglou D., Giassakis G., Piperidou H., Monastiriotis C., Maltezos E. (2005). Evaluation of a new indicator test for sudomotor function (Neuropad) in the diagnosis of peripheral neuropathy in type 2 diabetic patients. Exp. Clin. Endocrinol. Diabetes.

[214] Papanas N., Boulton A.J., Malik R.A., Manes C., Schnell O., Spallone V., Tentolouris N., Tesfaye S., Valensi P., Ziegler D. (2013). A simple new non-invasive sweat indicator test for the diagnosis of diabetic neuropathy. Diabet. Med..

[215] Papanas N., Ziegler D. (2014). New vistas in the diagnosis of diabetic polyneuropathy. Endocrine.

[216] Tentolouris N., Achtsidis V., Marinou K., Katsilambros N. (2008). Evaluation of the self-administered indicator plaster neuropad for the diagnosis of neuropathy in diabetes. Diabetes Care.

[217] Papanas N., Giassakis G., Papatheodorou K., Papazoglou D., Monastiriotis C., Christakidis D., Piperidou H., Maltezos E. (2007). Use of the new indicator test (Neuropad) for the assessment of the staged severity of neuropathy in type 2 diabetic patients. Exp. Clin. Endocrinol. Diabetes.

[218] Papanas N., Paschos P., Papazoglou D., Papatheodorou K., Paletas K., Maltezos E., Tsapas A. (2011). Accuracy of the neuropad test for the diagnosis of distal symmetric polyneuropathy in type 2 diabetes. Diabetes Care.

[219] Manes C., Papanas N., Exiara T., Katsiki N., Papantoniou S., Kirlaki E., Tsotoulidis S., Kefalogiannis N., Maltezos E. (2014). The indicator test Neuropad in the assessment of small and overall nerve fibre dysfunction in patients with type 2 diabetes: A large multicentre study. Exp. Clin. Endocrinol. Diabetes.

[220] Quattrini C., Jeziorska M., Tavakoli M., Begum P., Boulton A.J.M., Malik R.A. (2008). The Neuropad test: A visual indicator test for human diabetic neuropathy. Diabetologia.

[221] Hewitt N.D.B. The Neuropad test for the early detection of diabetic peripheral neuropathy. https://www.nice.org.uk/guidance/mtg38/documents/overview-of-assessment-report.

[222] Ziegler D., Papanas N., Roden M. (2011). Neuropad: Evaluation of three cut-off points of sudomotor dysfunction for early detection of polyneuropathy in recently diagnosed diabetes. Diabet. Med..

[223] NICE Neuropad for detecting preclinical diabetic peripheral neuropathy Medical technologies guidance (MTG38). https://www.nice.org.uk/guidance/mtg38.

[224] Goddard K., Pennington M., Kartha M.R., Macmillan T., Bunce C., Summers J.A., Keevil S., Chalkidou A. (2020). Complex clinical pathways: Assessing the value of a device for detecting diabetic peripheral neuropathy. Diabet. Foot J..

[225] Kubasch M.L., Kubasch A.S., Torres Pacheco J., Buchmann S.J., Illigens B.M.-W., Barlinn K., Siepmann T. (2017). Laser Doppler Assessment of Vasomotor Axon Reflex Responsiveness to Evaluate Neurovascular Function. Front. Neurol..

[226] Fromy B., Sigaudo-Roussel D., Gaubert-Dahan M.-L., Rousseau P., Abraham P., Benzoni D., Berrut G., Saumet J.L. (2010). Aging-Associated Sensory Neuropathy Alters Pressure-Induced Vasodilation in Humans. J. Investig. Dermatol..

[227] Caselli A., Rich J., Hanane T., Uccioli L., Veves A. (2003). Role of C-nociceptive fibers in the nerve axon reflex-related vasodilation in diabetes. Neurology.

[228] Caselli A., Spallone V., Marfia G.A., Battista C., Pachatz C., Veves A., Uccioli L. (2006). Validation of the nerve axon reflex for the assessment of small nerve fibre dysfunction. J. Neurol. Neurosurg. Psychiatry.

[229] Hamdy O., Abou-Elenin K., LoGerfo F.W., Horton E.S., Veves A. (2001). Contribution of nerve-axon reflex-related vasodilation to the total skin vasodilation in diabetic patients with and without neuropathy. Diabetes Care.

[230] Vas P.R., Rayman G. (2013). The rate of decline in small fibre function assessed using axon reflex-mediated neurogenic vasodilatation and the importance of age related centile values to improve the detection of clinical neuropathy. PLoS ONE.

[231] Vas P.R., Rayman G. (2013). Validation of the modified LDIFlare technique: A simple and quick method to assess C-fiber function. Muscle Nerve.

[232] Krishnan S.T., Rayman G. (2004). The LDIflare: A novel test of C-fiber function demonstrates early neuropathy in type 2 diabetes. Diabetes Care.

[233] Green A.Q., Krishnan S.T., Rayman G. (2009). C-fiber function assessed by the laser doppler imager flare technique and acetylcholine iontophoresis. Muscle Nerve.

[234] Green A.Q., Krishnan S., Finucane F.M., Rayman G. (2010). Altered C-fiber function as an indicator of early peripheral neuropathy in individuals with impaired glucose tolerance. Diabetes Care.

[235] Sharma S., Tobin V., Vas P.R.J., Malik R.A., Rayman G. (2018). The influence of age, anthropometric and metabolic variables on LDIFLARE and corneal confocal microscopy in healthy individuals. PLoS ONE.

[236] Krishnan S.T., Baker N.R., Carrington A.L., Rayman G. (2004). Comparative roles of microvascular and nerve function in foot ulceration in type 2 diabetes. Diabetes Care.

[237] Iqbal Z., Azmi S., Yadav R., Ferdousi M., Kumar M., Cuthbertson D.J., Lim J., Malik R.A., Alam U. (2018). Diabetic Peripheral Neuropathy: Epidemiology, Diagnosis, and Pharmacotherapy. Clin. Ther..

[238] Kalteniece A., Ferdousi M., Adam S., Schofield J., Azmi S., Petropoulos I., Soran H., Malik R.A. (2017). Corneal confocal microscopy is a rapid reproducible ophthalmic technique for quantifying corneal nerve abnormalities. PLoS ONE.

[239] Tavakoli M., Marshall A., Banka S., Petropoulos I.N., Fadavi H., Kingston H., Malik R.A. (2012). Corneal confocal microscopy detects small-fiber neuropathy in Charcot-Marie-Tooth disease type 1A patients. Muscle Nerve.

[240] Kemp H.I., Petropoulos I.N., Rice A.S.C., Vollert J., Maier C., Strum D., Schargus M., Peto T., Hau S., Chopra R. (2017). Use of Corneal Confocal Microscopy to Evaluate Small Nerve Fibers in Patients with Human Immunodeficiency Virus. JAMA Ophthalmol..

[241] Tavakoli M., Marshall A., Pitceathly R., Fadavi H., Gow D., Roberts M.E., Efron N., Boulton A.J., Malik R.A. (2010). Corneal confocal microscopy: A novel means to detect nerve fibre damage in idiopathic small fibre neuropathy. Exp. Neurol..

[242] Campagnolo M., Lazzarini D., Cacciavillani M., Fregona I., Bergamo F., Lonardi S., Midena E., Briani C. (2012). Corneal Confocal Microscopy in Patients with Chemotherapy-Induced Neuropathy. Neurology.

[243] Ferrari G., Gemignani F., Macaluso C. (2010). Chemotherapy-associated peripheral sensory neuropathy assessed using in vivo corneal confocal microscopy. Arch. Neurol..

[244] Torres R., Lopez-Moreno M., Muñoz M., Villoslada P., Sánchez-Dalmau B.F. Study of The Dynamics of Axonal Degeneration in Chemotherapy-Induced Neuropathy by In Vivo Corneal Confocal Microscopy. Proceedings of the 41st NANOS Annual Meeting.

[245] Tavakoli M., Malik R.A. (2011). Corneal confocal microscopy: A novel non-invasive technique to quantify small fibre pathology in peripheral neuropathies. JoVE.

[246] Asghar O., Petropoulos I.N., Alam U., Jones W., Jeziorska M., Marshall A., Ponirakis G., Fadavi H., Boulton A.J., Tavakoli M. (2014). Corneal confocal microscopy detects neuropathy in subjects with impaired glucose tolerance. Diabetes Care.

[247] Azmi S., Ferdousi M., Petropoulos I.N., Ponirakis G., Alam U., Fadavi H., Asghar O., Marshall A., Atkinson A.J., Jones W. (2015). Corneal Confocal Microscopy Identifies Small-Fiber Neuropathy in Subjects with Impaired Glucose Tolerance Who Develop Type 2 Diabetes. Diabetes Care.

[248] Chen X., Graham J., Dabbah M.A., Petropoulos I.N., Tavakoli M., Malik R.A. (2017). An Automatic Tool for Quantification of Nerve Fibers in Corneal Confocal Microscopy Images. IEEE Trans. Biomed. Eng..

[249] Chen X., Graham J., Petropoulos I.N., Ponirakis G., Asghar O., Alam U., Marshall A., Ferdousi M., Azmi S., Efron N. (2018). Corneal Nerve Fractal Dimension: A Novel Corneal Nerve Metric for the Diagnosis of Diabetic Sensorimotor Polyneuropathy. Investig. Ophthalmol. Vis. Sci..

[250] Dehghani C., Pritchard N., Edwards K., Vagenas D., Russell A.W., Malik R.A., Efron N. (2014). Natural history of corneal nerve morphology in mild neuropathy associated with type 1 diabetes: Development of a potential measure of diabetic peripheral neuropathy. Investig. Ophthalmol. Vis. Sci..

[251] Ishibashi F., Okino M., Ishibashi M., Kawasaki A., Endo N., Kosaka A., Uetake H. (2012). Corneal nerve fiber pathology in Japanese type 1 diabetic patients and its correlation with antecedent glycemic control and blood pressure. J. Diabetes Investig..

[252] Petropoulos I.N., Green P., Chan A.W., Alam U., Fadavi H., Marshall A., Asghar O., Efron N., Tavakoli M., Malik R.A. (2015). Corneal confocal microscopy detects neuropathy in patients with type 1 diabetes without retinopathy or microalbuminuria. PLoS ONE.

[253] Pritchard N., Edwards K., Russell A.W., Perkins B.A., Malik R.A., Efron N. (2015). Corneal confocal microscopy predicts 4-year incident peripheral neuropathy in type 1 diabetes. Diabetes Care.

[254] Bitirgen G., Ozkagnici A., Malik R.A., Kerimoglu H. (2014). Corneal nerve fibre damage precedes diabetic retinopathy in patients with type 2 diabetes mellitus. Diabet. Med..

[255] Efron N., Edwards K., Roper N., Pritchard N., Sampson G.P., Shahidi A.M., Vagenas D., Russell A., Graham J., Dabbah M.A. (2010). Repeatability of measuring corneal subbasal nerve fiber length in individuals with type 2 diabetes. Eye Contact Lens.

[256] Fadavi H., Tavakoli M., Foden P., Ferdousi M., Petropoulos I.N., Jeziorska M., Chaturvedi N., Boulton A.J.M., Malik R.A., Abbott C.A. (2018). Explanations for less small fibre neuropathy in South Asian versus European subjects with type 2 diabetes in the UK. Diabetes Metab. Res. Rev..

[257] Andersen S.T., Grosen K., Tankisi H., Charles M., Andersen N.T., Andersen H., Petropoulos I.N., Malik R.A., Jensen T.S., Karlsson P. (2018). Corneal confocal microscopy as a tool for detecting diabetic polyneuropathy in a cohort with screen-detected type 2 diabetes: ADDITION-Denmark. J. Diabetes Complicat..

[258] Khan A., Petropoulos I.N., Ponirakis G., Menzies R.A., Chidiac O., Pasquier J., Abi Khalil C., Talal T.K., Malik R.A. (2018). Corneal confocal microscopy detects severe small fiber neuropathy in diabetic patients with Charcot neuroarthropathy. J. Diabetes Investig..

[259] Malik R.A., Kallinikos P., Abbott C.A., van Schie C.H., Morgan P., Efron N., Boulton A.J. (2003). Corneal confocal microscopy: A non-invasive surrogate of nerve fibre damage and repair in diabetic patients. Diabetologia.

[260] Petropoulos I.N., Alam U., Fadavi H., Asghar O., Green P., Ponirakis G., Marshall A., Boulton A.J., Tavakoli M., Malik R.A. (2013). Corneal nerve loss detected with corneal confocal microscopy is symmetrical and related to the severity of diabetic polyneuropathy. Diabetes Care.

[261] Petropoulos I.N., Alam U., Fadavi H., Marshall A., Asghar O., Dabbah M.A., Chen X., Graham J., Ponirakis G., Boulton A.J. (2014). Rapid automated diagnosis of diabetic peripheral neuropathy with in vivo corneal confocal microscopy. Investig. Ophthalmol. Vis. Sci..

[262] Zhivov A., Peschel S., Schober H.-C., Stachs O., Baltrusch S., Bambi M.T., Kilangalanga J., Winter K., Kundt G., Guthoff R.F. (2015). Diabetic foot syndrome and corneal subbasal nerve plexus changes in congolese patients with type 2 diabetes. PLoS ONE.

[263] Stem M.S., Hussain M., Lentz S.I., Raval N., Gardner T.W., Pop-Busui R., Shtein R.M. (2014). Differential reduction in corneal nerve fiber length in patients with type 1 or type 2 diabetes mellitus. J. Diabetes Complicat..

[264] Tavakoli M., Quattrini C., Abbott C., Kallinikos P., Marshall A., Finnigan J., Morgan P., Efron N., Boulton A.J.M., Malik R.A. (2010). Corneal confocal microscopy: A novel noninvasive test to diagnose and stratify the severity of human diabetic neuropathy. Diabetes Care.

[265] Ferdousi M., Kalteniece A., Azmi S., Petropoulos I.N., Worthington A., D’Onofrio L., Dhage S., Ponirakis G., Alam U., Marshall A. (2020). Corneal confocal microscopy compared with quantitative sensory testing and nerve conduction for diagnosing and stratifying the severity of diabetic peripheral neuropathy. BMJ Open Diabetes Res. Care.

[266] Rosenberg M.E., Tervo T.M.T., Immonen I.J., Müller L.J., Grönhagen-Riska C., Vesaluoma M.H. (2000). Corneal Structure and Sensitivity in Type 1 Diabetes Mellitus. Investig. Ophthalmol. Vis. Sci..

[267] Lewis E.J.H., Lovblom L.E., Ferdousi M., Halpern E.M., Jeziorska M., Pacaud D., Pritchard N., Dehghani C., Edwards K., Srinivasan S. (2020). Rapid Corneal Nerve Fiber Loss: A Marker of Diabetic Neuropathy Onset and Progression. Diabetes Care.

[268] Dehghani C., Russell A.W., Perkins B.A., Malik R.A., Pritchard N., Edwards K., Shahidi A.M., Srinivasan S., Efron N. (2016). A rapid decline in corneal small fibers and occurrence of foot ulceration and Charcot foot. J. Diabetes Complicat..

[269] Mehra S., Tavakoli M., Kallinikos P.A., Efron N., Boulton A.J.M., Augustine T., Malik R.A. (2007). Corneal Confocal Microscopy Detects Early Nerve Regeneration After Pancreas Transplantation in Patients with Type 1 Diabetes. Diabetes Care.

[270] Tavakoli M., Ferdousi M., Petropoulos I.N., Morris J., Pritchard N., Zhivov A., Ziegler D., Pacaud D., Romanchuk K., Perkins B.A. (2015). Normative values for corneal nerve morphology assessed using corneal confocal microscopy: A multinational normative data set. Diabetes Care.

[271] Perkins B.A., Lovblom L.E., Bril V., Scarr D., Ostrovski I., Orszag A., Edwards K., Pritchard N., Russell A., Dehghani C. (2018). Corneal confocal microscopy for identification of diabetic sensorimotor polyneuropathy: A pooled multinational consortium study. Diabetologia.

[272] Ostrovski I., Lovblom L.E., Farooqi M.A., Scarr D., Boulet G., Hertz P., Wu T., Halpern E.M., Ngo M., Ng E. (2015). Reproducibility of In Vivo Corneal Confocal Microscopy Using an Automated Analysis Program for Detection of Diabetic Sensorimotor Polyneuropathy. PLoS ONE.

[273] Dabbah M.A., Graham J., Petropoulos I., Tavakoli M., Malik R.A. (2010). Dual-model automatic detection of nerve-fibres in corneal confocal microscopy images. Med. Image Comput. Comput. Assist. Interv..

[274] Dabbah M.A., Graham J., Petropoulos I.N., Tavakoli M., Malik R.A. (2011). Automatic analysis of diabetic peripheral neuropathy using multi-scale quantitative morphology of nerve fibres in corneal confocal microscopy imaging. Med. Image Anal..

[275] Williams B.M., Borroni D., Liu R., Zhao Y., Zhang J., Lim J., Ma B., Romano V., Qi H., Ferdousi M. (2020). An artificial intelligence-based deep learning algorithm for the diagnosis of diabetic neuropathy using corneal confocal microscopy: A development and validation study. Diabetologia.

[276] Tavakoli M., Kallinikos P., Iqbal A., Herbert A., Fadavi H., Efron N., Boulton A.J.M., Malik R.A. (2011). Corneal confocal microscopy detects improvement in corneal nerve morphology with an improvement in risk factors for diabetic neuropathy. Diabet. Med. J. Br. Diabet. Assoc..

[277] Kural M.A., Andersen S.T., Andersen N.T., Andersen H., Charles M., Finnerup N.B., Jensen T.S., Tankisi H. (2019). The utility of a point-of-care sural nerve conduction device for detection of diabetic polyneuropathy: A cross-sectional study. Muscle Nerve.

[278] Dyck P.J., Albers J.W., Andersen H., Arezzo J.C., Biessels G.-J., Bril V., Feldman E.L., Litchy W.J., O’Brien P.C., Russell J.W. (2011). Diabetic polyneuropathies: Update on research definition, diagnostic criteria and estimation of severity. Diabetes Metab. Res. Rev..

[279] Krieger S.-M., Reimann M., Haase R., Henkel E., Hanefeld M., Ziemssen T. (2018). Sudomotor Testing of Diabetes Polyneuropathy. Front. Neurol..

[280] Yajnik C.S., Kantikar V.V., Pande A.J., Deslypere J.P. (2012). Quick and simple evaluation of sudomotor function for screening of diabetic neuropathy. ISRN Endocrinol..

[281] Carbajal-Ramirez A., Hernandez-Dominguez J.A., Molina-Ayala M.A., Rojas-Uribe M.M., Chavez-Negrete A. (2019). Early identification of peripheral neuropathy based on sudomotor dysfunction in Mexican patients with type 2 diabetes. BMC Neurol..

[282] Binns-Hall O., Selvarajah D., Sanger D., Walker J., Scott A., Tesfaye S. (2018). One-stop microvascular screening service: An effective model for the early detection of diabetic peripheral neuropathy and the high-risk foot. Diabet. Med..

[283] Eranki V.G., Santosh R., Rajitha K., Pillai A., Sowmya P., Dupin J., Calvet J.H. (2013). Sudomotor function assessment as a screening tool for microvascular complications in type 2 diabetes. Diabetes Res. Clin. Pract..

[284] Liatis S., Marinou K., Tentolouris N., Pagoni S., Katsilambros N. (2007). Usefulness of a new indicator test for the diagnosis of peripheral and autonomic neuropathy in patients with diabetes mellitus. Diabet. Med..

[285] Papanas N., Papatheodorou K., Papazoglou D., Monastiriotis C., Christakidis D., Maltezos E. (2008). A comparison of the new indicator test for sudomotor function (Neuropad) with the vibration perception threshold and the clinical examination in the diagnosis of peripheral neuropathy in subjects with type 2 diabetes. Exp. Clin. Endocrinol. Diabetes.

[286] Bilen H., Atmaca A., Akcay G. (2007). Neuropad indicator test for diagnosis of sudomotor dysfunction in type 2 diabetes. Adv. Ther..

[287] Freitas C., Carvalho A., Melo-Rocha G., Amaral C., Pinto S., Guimaraes R., Neto H., Suascun J., Muras J., Goncalves I. (2009). The Neuropad test in the screening of peripheral neuropathy in diabetic patients. Acta Med. Port..

[288] Ishibashi F., Kojima R., Kawasaki A., Yamanaka E., Kosaka A., Uetake H. (2014). Correlation between sudomotor function, sweat gland duct size and corneal nerve fiber pathology in patients with type 2 diabetes mellitus. J. Diabetes Investig..

[289] Kamenov Z.A., Petrova J.J., Christov V.G. (2010). Diagnosis of diabetic neuropathy using simple somatic and a new autonomic (neuropad) tests in the clinical practice. Exp. Clin. Endocrinol. Diabetes.

[290] Ponirakis G., Petropoulos I.N., Fadavi H., Alam U., Asghar O., Marshall A., Tavakoli M., Malik R.A. (2014). The diagnostic accuracy of Neuropad for assessing large and small fibre diabetic neuropathy. Diabet. Med..

[291] Ahmed A., Bril V., Orszag A., Paulson J., Yeung E., Ngo M., Orlov S., Perkins B.A. (2012). Detection of diabetic sensorimotor polyneuropathy by corneal confocal microscopy in type 1 diabetes: A concurrent validity study. Diabetes Care.

[292] Chen X., Graham J., Dabbah M.A., Petropoulos I.N., Ponirakis G., Asghar O., Alam U., Marshall A., Fadavi H., Ferdousi M. (2015). Small nerve fiber quantification in the diagnosis of diabetic sensorimotor polyneuropathy: Comparing corneal confocal microscopy with intraepidermal nerve fiber density. Diabetes Care.

[293] Edwards K., Pritchard N., Vagenas D., Russell A., Malik R.A., Efron N. (2014). Standardizing corneal nerve fibre length for nerve tortuosity increases its association with measures of diabetic neuropathy. Diabet. Med..

[294] Ponirakis G., Fadavi H., Petropoulos I.N., Azmi S., Ferdousi M., Dabbah M.A., Kheyami A., Alam U., Asghar O., Marshall A. (2015). Automated Quantification of Neuropad Improves Its Diagnostic Ability in Patients with Diabetic Neuropathy. J. Diabetes Res..

[295] Tavakoli M., Begum P., McLaughlin J., Malik R.A. (2015). Corneal confocal microscopy for the diagnosis of diabetic autonomic neuropathy. Muscle Nerve.

[296] Pritchard N., Dehghani C., Edwards K., Burgin E., Cheang N., Kim H., Mikhaiel M., Stanton G., Russell A.W., Malik R.A. (2015). Utility of Assessing Nerve Morphology in Central Cornea Versus Whorl Area for Diagnosing Diabetic Peripheral Neuropathy. Cornea.

[297] Wang M., Zhang C., Zuo A., Li L., Chen L., Hou X. (2020). Diagnostic utility of corneal confocal microscopy in type 2 diabetic peripheral neuropathy. J. Diabetes Investig..

[298] Ferdousi M., Kalteniece A., Azmi S., Petropoulos I.N., Ponirakis G., Alam U., Asghar O., Marshall A., Fullwood C., Jeziorska M. (2021). Diagnosis of Neuropathy and Risk Factors for Corneal Nerve Loss in Type 1 and Type 2 Diabetes: A Corneal Confocal Microscopy Study. Diabetes Care.

